# Frequent inactivating mutations of *STAG2* in bladder cancer are associated with low tumour grade and stage and inversely related to chromosomal copy number changes

**DOI:** 10.1093/hmg/ddt589

**Published:** 2013-11-22

**Authors:** Claire F. Taylor, Fiona M. Platt, Carolyn D. Hurst, Helene H. Thygesen, Margaret A. Knowles

**Affiliations:** 1 Cancer Research UK Centre Genomics Facility,; 2 Sections of Experimental Oncology,; 3 Epidemiology and Biostatistics, Leeds Institute of Cancer and Pathology, St James’s University Hospital, Beckett Street, Leeds LS9 7TF, UK

## Abstract

Inactivating mutations of *STAG2* have been reported at low frequency in several cancers. In glioblastoma, the function of STAG2 has been related to maintenance of euploidy via its role in the cohesin complex. In a screen of a large series of bladder tumours and cell lines, we found inactivating mutations (nonsense, frameshift and splicing) in 67 of 307 tumours (21.8%) and 6 of 47 cell lines. Thirteen missense mutations of unknown significance were also identified. Inactivating mutation was associated with low tumour stage (*P* = 0.001) and low grade (*P* = 0.0002). There was also a relationship with female patient gender (*P* = 0.042). Examination of copy number profiles revealed an inverse relationship of mutation with both fraction of genome altered and whole chromosome copy number changes. Immunohistochemistry showed that in the majority of cases with inactivating mutations, STAG2 protein expression was absent. Strikingly, we identified a relatively large subset of tumours (12%) with areas of both positive and negative immunoreactivity, in only four of which a potentially function-altering mutation was detected. Regions of differential expression were contiguous and showed similar morphological phenotype in all cases. Microdissected positive and negative areas from one tumour showed an inactivating mutation to be present only in the negative area, suggesting intra-tumoral sub-clonal genomic evolution. Our findings indicate that loss of STAG2 function plays a more important role in non-invasive than that in muscle-invasive bladder cancer and suggest that cohesin complex-independent functions are likely to be important in these cases.

## INTRODUCTION

Inactivating mutations in the cohesin complex component *STAG2* have been reported in 5.9% of glioblastoma and at lower frequency in melanoma and Ewing's sarcoma (1) and several other cancer types [COSMIC; cancer.sanger.ac.uk/cancergenome/projects/cosmic/].

Cohesin is a four-subunit ring-shaped complex, comprised in mammalian cells of SMC1, SMC3, RAD21 and STAG1 or STAG2. The complex mediates cohesion between sister chromatids following DNA replication to ensure correct chromosomal segregation. Loading of cohesin onto chromatin occurs during the G1 phase of the cell cycle, and the complex becomes tightly closed during DNA replication to maintain chromatid cohesion. During prophase of mitosis, all cohesin apart from that at the centromeres is removed. Finally, in anaphase, centromeric cohesin is removed to allow chromosome segregation (2). In vertebrates, cohesin complexes containing STAG1 and STAG2 fulfil distinct functions in chromatid cohesion, STAG1-cohesin mediating telomere cohesion and STAG2-cohesin mediating centromeric cohesion. Loss of either STAG1 or STAG2 has been associated with the generation of aneuploidy in mammalian cells. For example, STAG1-deficient mouse embryo fibroblasts show increased aneuploidy (3), and functional assays in glioblastoma cell lines have linked loss of STAG2 expression to chromatid cohesion defects and aneuploidy (1). These data suggest that STAG2 may function as a ‘caretaker’ tumour suppressor gene, leading to chromosomal instability when inactivated.

In addition to the well-documented functions during cell division, cohesin also plays a role as an organizer of interphase chromatin. STAG1-cohesin has been most studied in this context. It has a role in restricting γH2AX accumulation at double-strand breaks to allow continued expression of neighbouring genes, and it co-localizes at many sites with CCCTC-binding factor (CTCF) and other transcriptional regulators, where it plays a role in regulating gene expression. Evidence to date suggests that these roles are mainly related to STAG1-cohesin [reviewed in (4)] and indeed, repair of inactivating STAG2 mutation to generate normal expression in glioblastoma cells was recently reported to have no significant effect on the transcriptional profile, suggesting that the role of STAG2 may be restricted to its functions in chromatid cohesion (1). However, as STAG2 is more abundant than STAG1 and can directly bind to CTCF (5), and as CTCF interactions are cell context-specific, non-chromatid cohesion-related effects of STAG2 loss appear likely in some cell types.

The COSMIC database (http://cancer.sanger.ac.uk/cancergenome/projects/cosmic/) lists 11 missense, splicing or nonsense mutations in *STAG2* in bladder cancer (25.7.13, date last accessed)*.* Eight of these are in 104 muscle-invasive tumour samples included in the TCGA study of advanced bladder cancer (http://cancergenome.nih.gov/cancersselected/invasiveurothelialbladder), and three in muscle-invasive or superficially invasive tumours from the study of Gui *et al*. (6). Such tumours are commonly highly aneuploid. However, bladder cancers comprise at least two distinct groups, with marked differences in molecular profiles. The majority of bladder tumours at presentation are low-grade non-invasive tumours, most of which are genomically stable with minimal aneuploidy (7,8). During exome sequencing of a small panel of such non-invasive bladder tumours (stage Ta), we identified several inactivating mutations in *STAG2* (Hurst *et al.*, unpublished data). As these samples showed minimal copy number alterations by array-based CGH, loss of a chromatid-related cohesin function was unexpected. Therefore, we have carried out a mutation analysis of the entire coding sequence of *STAG2* and assessed STAG2 protein expression in a large panel of bladder cancers and cell lines. Here we report the mutation spectrum and the relationship of loss of STAG2 expression with gender, tumour grade, stage and chromosomal stability. Our data suggest that loss of STAG2 function plays a more important role in non-invasive than in muscle-invasive bladder cancer and that cohesin complex-independent functions are likely to be important in these cases.

## RESULTS

### Mutation of STAG2 is frequent in urothelial carcinoma

*STAG2* has 33 coding exons, which encode a 141-kD protein. It is alternatively spliced both in the 5′ UTR and in the coding region by inclusion or exclusion of exon 33. We scanned the entire coding sequence using high-resolution melting (HRM) in 307 bladder tumours and 47 tumour cell lines. *STAG2* maps to the X chromosome (Xq25) and is present as only a single copy in males. As HRM relies on the presence of wild-type sequence to generate mutant/wild-type heteroduplexes with aberrant melting profiles, mutations in pure tumour DNA from males may not be detected. Therefore, a workflow was devised (Supplementary Material, Fig. S1) that incorporated re-analysis following ‘spiking’ with normal DNA of all male samples that did not show aberrant melting profiles on first analysis. Cell lines were all analysed in the presence of added normal DNA.

In the initial screening, variants were identified in 69 tumours and 8 cell lines (92-1, 94-10, HCV29, U-BLC1, UM-UC3, UM-UC14, VM-CUB-1 and VM-CUB-3). One hundred and sixty-eight apparently wild-type samples from male patients were then re-screened in the presence of normal DNA, and this revealed an additional 10 variants. Variants in tumour tissues were confirmed as somatic by comparison with constitutional DNA from the same patient. Four tumours and one cell line contained two variants. In tumours, we identified 27 nonsense mutations. In tumours 990 and 1474, the change to the DNA sequence is a short deletion, which results in immediate termination of the reading frame without any frameshifted sequence. Twenty-two tumours had insertion/deletion mutations, which resulted in a frameshift, and one tumour had an in-frame deletion located within the STAG domain. In cell lines, one nonsense and one frameshift mutation were detected, each caused by small indels (Table 1; Supplementary Material, Table S1).

**Table 1.** Variants identified in STAG2 in bladder tumours and cell lines

Sample	Position of variant in transcript^a^	Predicted effect at primary amino acid sequence level	Effect^b^
Tumour			
595	c.50 C>G	p.S17*	Nonsense
1052	c.256 G>T	p.E86*	Nonsense
1145	c.367 C>T	p.Q123*	Nonsense
1436	c.367 C>T	p.Q123*	Nonsense
359	c.385+1 G>T	p.?	Splice (presumed)
1046	c.386-8 A>G	p.?	Splice (presumed)
1167	c.407_414 del8	p.F136fs	Frameshift
1231	c.462 G>C	p.?	Splice
315	c.499 C>T	p.Q167*	Nonsense
1025	c.605 C>A	p.S202*	Nonsense
991	c.625_642 del18	p.D209_A214del	In-frame deletion
385	c.739 C>T	p.Q247*	Nonsense
985	c.775 C>T	p.R259*	Nonsense
868	c.809 dupA	p.R271fs	Frameshift
1332	c.832 C>T	p.Q278*	Nonsense
1383	c.1017+1_1017+22 delinsCATCTTAC	p.?	Splice
1474	c.1036 A>C; c.2707_2710 del4	p.K346Q; p.T903*	Missense; nonsense
811	c.1044 dupT	p.T349fs	Frameshift
717	c.1071_1072 delCA	p.N357fs	Frameshift
582	c.1113 C>G	p.F371L	Missense
1473	c.1117-34 C>T	p. (=)	No effect
1175	c.1196+4 A>G	p.?	Splice
494	c.1347_1351 del5	p.R451fs	Frameshift
1696	c.1354_1387 del34	p.G452fs	Frameshift
1116	c.1374 delC	p.N459fs	Frameshift
322	c.1401 delinsTG	p.F468fs	Frameshift
1135	c.1401 delinsTG	p.F468fs	Frameshift
987	c.1417-7 A>G	p.?	Splice
865	c.1444 dupG	p.D482fs	Frameshift
1063	c.1505 del T	p.L502fs	Frameshift
1072	c.1534+24 T>A	p.=	No effect
1345	c.1535-40_1566 del72	p.?	Splice
1415	c.1549 C>T	p.Q517*	Nonsense
454	c.1580_1581 dupGT	p.T528fs	Frameshift
869	c.1591 delC	p.Q531fs	Frameshift
1298	c.1693 G>T	p.E565*	Nonsense
1017	c.1811 G>T	p.R604L	Missense
1028	c.1815A>C; c.2655 C>T	p.L605F; p. (=)	Missense; synonymous
417	c.1866_1884 del19	p.K622fs	Frameshift
1465	c.1908 C>A	p.Y636*	Nonsense
525	c.1958 C>G	p.S653*	Nonsense
1338	c.1988 A>G	p.D663G	Missense
961	c.2026-1 G>T	p.?	Splice
1529	c.2056 C>T	p.Q686*	Nonsense
736	c.2097-3 C>G	p.?	Splice
1214	c.2097-2 A>G	p.?	Splice (presumed)
1210	c.2097-1 G>A	p.?	Splice
271	c.2201 T>C	p.L734P	Missense
983	c.2207_2211 del5	p.C736fs	Frameshift
990	c.2208_2219 del12	p.C736*	Nonsense
339	c.2212 C>T	p.H738Y	Missense
536	c.2229 G>A	p.W743*	Nonsense
945	c.2256_2265+16 del26	p.?	Splice
1185	c.2279 delinsCT	p.R760fs	Frameshift
1053	c.2308 C>T	p.Q770*	Nonsense
860	c.2533 G>A	p.? & p.D845N	Splice and missense
1142	c.2534-4_2550 del21	p.?	Splice
1079	c.2542 C>T	p.Q848*	Nonsense
670	c.2775+2 delT	p.?	Splice
1083	c.2822 C>G	p.S941*	Nonsense
1073	c.2848 G>A	p.E950K	Missense
989	c.2888 A>G	p.Q963R	Missense
1273	c.2924+1 G>A	p.?	Splice
1111	c.2977 C>T; c.3085 C>T	p.P993S; p.Q1029*	Missense; nonsense
500	c.3054-15 C>T; c.3085 C>T	p. (=); p.Q1029*	No effect; nonsense
1294	c.3063 C>G	p.Y1021*	Nonsense
140	c.3074 delT	p.F1025fs	Frameshift
137	c.3085 C>T	p.Q1029*	Nonsense
511	c.3097 C>T	p.R1033*	Nonsense
1434	c.3133 C>T	p.R1045*	Nonsense
643	c.3143 delT	p.L1048fs	Frameshift
397	c.3153_3154 dupTG	p.G1052fs	Frameshift
1335	c.3190_3191 delinsT	p.S1065fs	Frameshift
578	c.3278-3_3296 del22	p.?	Splice
1081	c.3364 dupA	p.T1122fs	Frameshift
529	c.3407_3410 dup4	p.S1137fs	Frameshift
1324	c.3448 C>G	p.Q1150E	Missense
1094	c.3644 C>G	p.S1215*	Nonsense
1172	c.3772 A>G	p.M1258V	Missense
Cell line			
UM-UC14	c.-32 G>A; c.2026-1 G>T	p.?; p.?	Unknown effect; splice
VM-CUB-3	c.289-2 A>G	p.?	Splice
U-BLC1	c.1731+28 A>C	p.=	No effect
HCV29	c.1756 dupA	p.T586fs	Frameshift
94-10	c.2097-2 A>G	p.?	Splice
VM-CUB-1	c.2674-6_2686 dup	p.?	Splice
UM-UC3	c.2947_2948 delinsT	p.K983*	Nonsense
92-1	c.3787 C>G	p.L1263V	Missense

^a^NM_001042749 (numbered with A of ATGi as nucleotide 1).

^b^Splice mutations supported by RNA evidence unless indicated otherwise.

Mutations at consensus splice sites were found in several tumours and cell lines. These comprised eight point mutations in invariant nucleotides, two in conserved nucleotides and six deletions that encompassed invariant nucleotides. One cell line, VM-CUB-1, had a duplication of the 3′ splice site of exon 28. Several samples had somatic intronic variants that were not within splice site sequences. In each case, the potential for an effect on splicing was assessed using the SSPNN splice site prediction tool (http://www.fruitfly.org/seq_tools/splice.html). No plausible effect was identified for intronic variants in tumours 1473 and 500 or cell line U-BLC1, so these variants were regarded as passenger variants and not considered further. The variants in tumours 1046, 1072 and 987 were regarded as having potential to disrupt splicing. Tumours 1231 and 860 had substitutions in the final nucleotides of exons 8 and 26, respectively. This position is the exonic part of the consensus 5′ splice site, so these mutations were considered for a role in splicing even though they would be missense mutations by their effect on the primary amino acid sequence. SSPNN in each case suggested that there would be a decrease in the strength of the splice site in the presence of the mutation.

**Figure 1.**
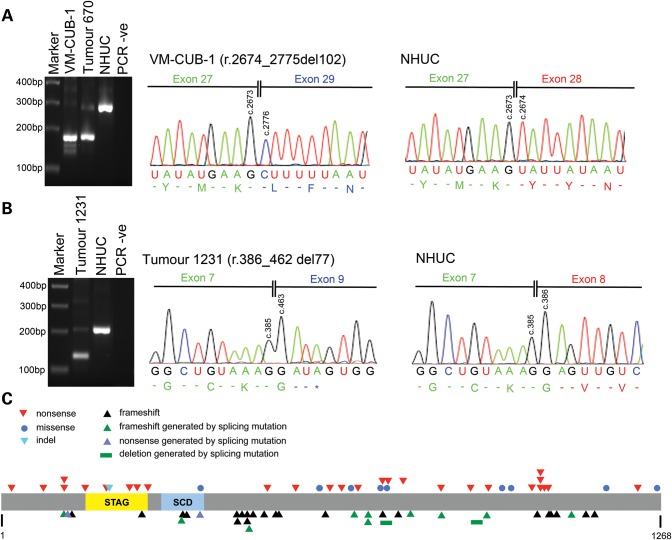
STAG2 mutations in bladder cancer samples. STAG2 splice site mutations in (**A**) cell line VM-CUB-1 and (**B**) bladder tumour 1231. Left-hand panels in (A) and (B) show aberrantly sized bands generated by RT-PCR, and right-hand panels show nucleotide sequences of aberrant RT-PCR products illustrating the effects of splicing mutations at the RNA level. (A) PCR was performed on VM-CUB-1 cDNA using primers in exons 27 and 29. A PCR product 102 bp smaller than that generated using cDNA from normal human urothelial cells (NHUC) was observed for VM-CUB-1. Sanger sequencing of RT-PCR products showed that this aberrantly sized band lacked exon 28. Bladder tumour 670 carries a splice site mutation that is also predicted to result in skipping of exon 28, and this sample generated a PCR product similar in size to that observed for VM-CUB-1. Sequencing confirmed that this PCR product also lacked exon 28 (data not shown). (B) PCR was performed on cDNA from bladder tumour 1231 using primers in exons 7 and 9. A PCR product 77 bp smaller than that generated using cDNA from NHUC was observed for tumour 1231, and sequencing confirmed that the aberrantly sized PCR product lacked exon 8. A detailed description of splice site mutations in all samples is given in Supplementary Material, text. (**C**) Diagram of STAG2 protein showing position of somatic nonsense, missense, splicing, frameshift and indel mutations identified in bladder tumours. STAG and SMC (structural maintenance of chromosomes) domains are also shown.

Of the 22 samples with mutations that had a potential for a role in splicing, RNA was available for 19. RT-PCR was carried out using primers located within exons flanking the exon whose splice sites were affected by the mutation. RT-PCR products were analysed by agarose gel electrophoresis and Sanger sequencing (Fig. 1A and B; Supplementary Material, Table S4; Supplementary Material, text). A role in splicing was confirmed for all except that in tumour 1072, which is therefore regarded as a passenger variant. The effects on splicing were exon skipping and/or use of local cryptic splice sites except in one tumour (1345) in which no STAG2 RT-PCR products could be detected, although control RT-PCR showed that there was adequate RNA in the sample. The consequences of these mutations, incorporating knowledge from RNA analysis, are given in Supplementary Material, Table S4. In three samples, tumours 670 and 945 and cell line VM-CUB-1, the consequence is in-frame skipping of an exon (Fig. 1A). All other splicing mutants resulted in premature truncation apart from the exonic variant in tumour 860, which produced both an exon-skipped product (and thus prematurely truncated) and also a product in which the mutant exon was included. Thus, this mutation may operate at both splicing and missense levels. Taken together, these data indicate that 67 of 307 tumours examined (21.8%) contained mutations that are predicted to adversely affect STAG2 function (truncation with or without frameshift or deletion) (Table 1).

**Figure 2.**
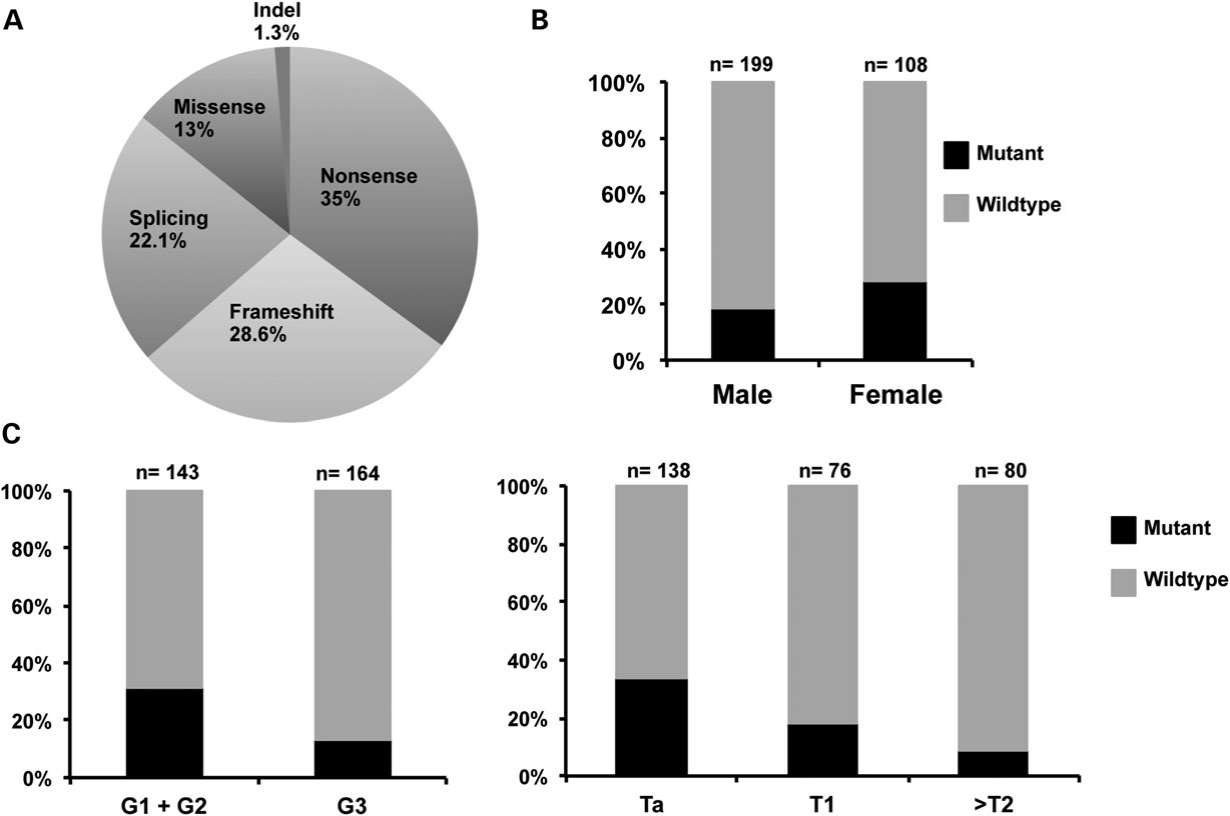
*STAG2* mutation types and relationship to patient gender, tumour grade and stage. (**A**) Pie chart showing distribution of mutation types. (**B**) Distribution of mutations in relation to gender. (**C**) Distribution of mutations in relation to tumour grade and stage.

Twelve tumours and one cell line had missense mutations, the functional effect of which are unknown. All were at amino acid residues that are invariant in all vertebrate species examined (*Homo sapiens, Macaca mulatta, Canis lupus, Bos taurus, Rattus norvegicus, Mus musculus, Gallus gallus* and *Danio rerio*). Two missense mutations were present in tumours that also contained nonsense mutations (tumours 1474 and 1111). The location of mutations is shown in Fig. 1C and their distribution by type in Fig. 2A.

### *STAG2* mutation is associated with low tumour grade and stage and with female gender

We assessed the relationship of *STAG2* mutation with tumour grade, stage and patient gender. As there is uncertainty about the effect of missense mutations on protein function, these analyses were all carried out twice, first with only inactivating mutations included and then including all mutations. There was a significant association with low tumour grade (Fisher's exact test, *P* = 0.0002 and *P* = 0.0023, respectively) and with lower tumour stage (chi-square test, *P* = 0.001 and *P* = 0.044, respectively) (Fig. 2C). Inactivating mutation was also significantly associated with female gender (Fisher's exact test, *P* = 0.042) (Fig. 2B) but not when missense mutations were included in the analysis (Fisher's exact test, *P* = 0.073). We examined the relationship of mutation status with disease recurrence (median follow-up, 81 months). No significant relationships were uncovered when inactivating mutations only or all mutations were included [hazard ratio = 1.14 (*P* = 0.53) and 1.34 (*P* = 0.15) for all tumours, 1.04 (*P* = 0.92) and 1.32 (*P* = 0.49) for stage T1+ ≥T2 tumours and 1.25 (*P* = 0.38) and 1.46 (*P* = 0.13) for stage Ta tumours].

We also tested for association between *STAG2* mutation and *FGFR3*, *PIK3CA, TP53* and RAS gene mutation (7). These tests were carried out both with and without inclusion of *STAG2* missense mutations. In both cases, mutation was significantly associated with *FGFR3* mutation (Fisher's exact test, *P* = 1.16 × 10^−5^ and 8.82 × 10^−7^, respectively) and with *PIK3CA* mutation (*P* = 0.0048 and 0.0067, respectively) but not with RAS gene mutation (*P* = 0.507 and 0.818, respectively). Mutations were significantly associated with wild-type status for *TP53* only when missense mutations of *STAG2* were not considered (*P* = 0.032). In the cell lines that were found to contain a mutation, mutation status for *FGFR3, PIK3CA,* RAS genes and *TP53* was available but the same relationships were not found. Two of these lines contain an *FGFR3* mutation (94-10 and UM-UC14), one contains a *KRAS* mutation (UM-UC3), two contain mutations in *PIK3CA* (VM-CUB-1 and VM-CUB-3) and six have mutations in *TP53* (all except 94-10 and HCV29) (Supplementary Material, Table S5). This high frequency of *TP53* mutation differs from that in the tumour population in which we detected *STAG2* mutations. As the majority of available urothelial carcinoma (UC) cell lines are derived from invasive tumours, including the five *STAG2* mutant cell lines for which stage and/or grade information is available, they are not representative of the spectrum of UC in general.

### STAG2 mutation is not associated with chromosomal copy number alterations

**Figure 3.**
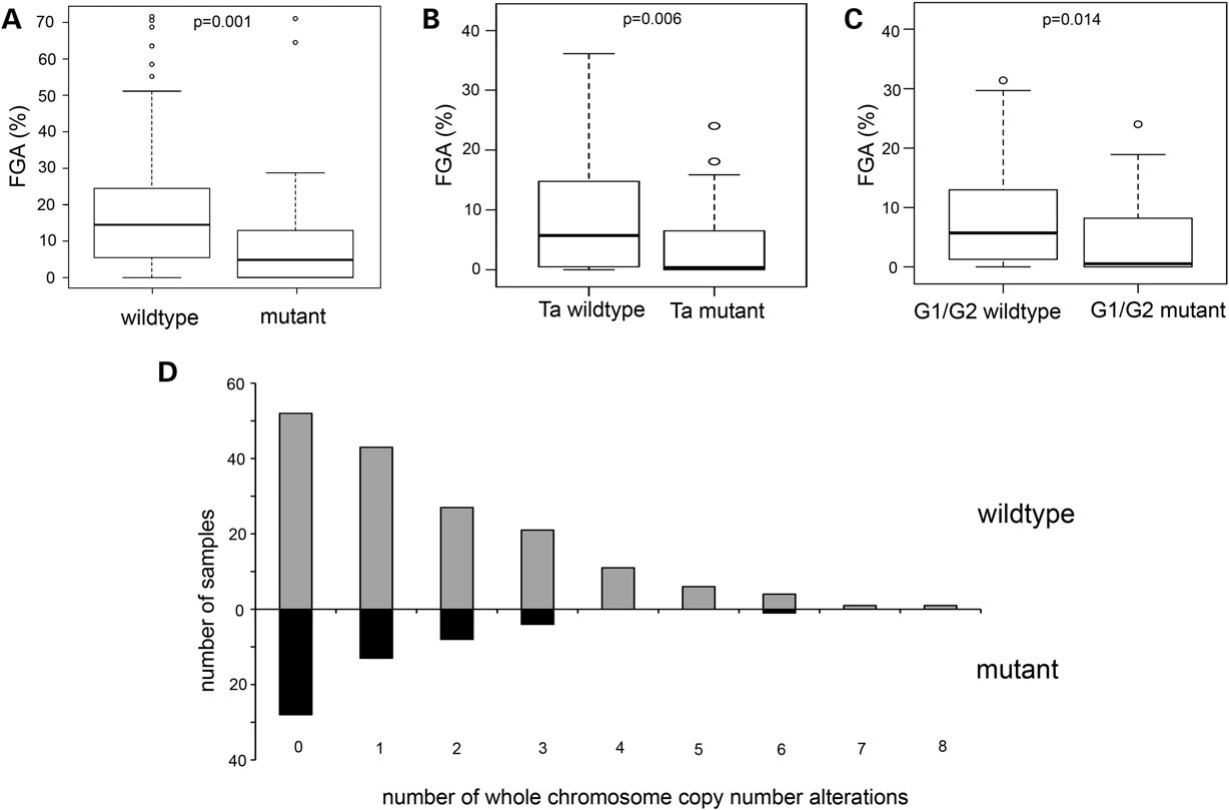
Relationship of *STAG2* mutation with copy number alterations. Box plots showing the relationship between FGA and mutation status in (**A**) tumours of all stages and grades, (**B**) stage Ta tumours and (**C**) grade 1/2 tumours. The top and bottom of the boxes represent the 75 and 25% percentiles, respectively. Whiskers represent 1.5 times the upper and lower quartile limits, unless this exceeds the range of the data in which case whiskers extend from maximum to minimum values. Black lines represent medians. Outliers are shown as open circles. (**D**) Histogram showing whole chromosome copy number alterations in *STAG2* wild-type (grey bars) and mutant (black bars) bladder tumours.

Previously, loss of STAG2 function was reported to be associated with generation of aneuploidy (1). As low-grade, low-stage bladder tumours are frequently near-diploid, the association of inactivating mutation with these features was not anticipated. BAC array CGH data were available for 220 tumours [(7) and unpublished data]. To gain a more accurate assessment of the relationship of *STAG2* mutation with genomic instability, we tested the relationship of mutation with genome-wide copy number alterations measured as fraction of genome altered (FGA; percentage of clones on the array that reported significantly altered copy number). Lower mean FGA was found in *STAG2* mutant tumours (*t*-test, *P* < 0.001) (Fig. 3A). When subdividing by stage or grade, significantly lower FGA was found in stage Ta (*t*-test, *P* = 0.006) and grade 2 (*t*-test, *P* = 0.014) *STAG2* mutant tumours (Fig. 3B and C). To provide a better measure of aneuploidy, we examined the number of whole chromosome copy number alterations in this dataset (Supplementary Material, Table S1). This measure was also significantly lower in tumours with *STAG2* mutation (Mann–Whitney *U*-test, *P* < 0.002) (Fig. 3D). When subdividing by tumour stage, this was also significantly lower in Ta and grade 1/2 tumours (*P* = 0.03; *P* = 0.039).

### Intra-tumour heterogeneity in STAG2 protein expression

**Figure 4.**
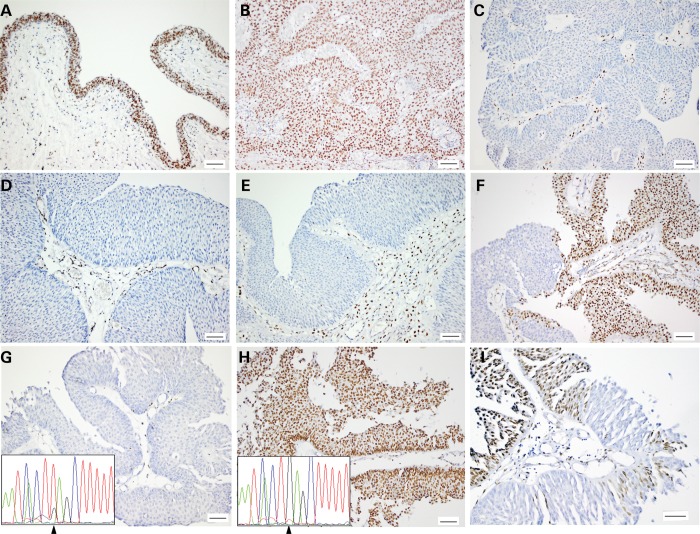
STAG2 expression detected by immunohistochemistry in normal ureteric urothelium and urothelial carcinoma. (**A**) Normal ureter. (**B**) Urothelial carcinoma with wild-type *STAG2* sequence and normal protein expression. (**C**) Tumour 643 (p.L1048fs) showing absence of expression in tumour cells and positive staining in stroma. (**D**) Tumour 511 (p.R1033*). (**E**) Tumour 961 with splicing mutation (c.2026-1 G>T). (**F**) Tumour 866 with chimeric staining pattern. (**G–I**) Sections from tumour 1298. (G) Areas with absence of expression containing inactivating mutation (c.1693 G>T; p.E565*; arrow). (H) Area of positive staining containing wild-type sequence. (I) Detail showing contiguity of positive and negative staining.

Immunohistochemistry with a mouse monoclonal antibody raised against recombinant human STAG2 was used to assess STAG2 protein expression in all tumours for which formalin-fixed, paraffin-embedded (FFPE) blocks were available (*n* = 120). Of these, 15 had been shown to contain nonsense or frameshift mutations, 6 contained mutations shown or predicted to affect splicing, 1 had an intronic variant predicted to have no effect and 5 contained missense mutations (Supplementary Material, Table S1). The specificity of the antibody used has been confirmed previously (1). Normal bladder and ureter used as positive controls showed strong nuclear staining in all urothelial cell layers (Fig. 4), and all tissues showed strong nuclear staining in stromal cells, which provided an internal positive control.

Of the 15 tumours with nonsense or frameshift mutations, 11 showed no detectable STAG2 protein, 2 showed positive staining and 2 contained both positive and negative areas (Supplementary Material, Table S1). DNA was extracted from whole FFPE sections of the two tumours with positive staining and frameshift (454) or nonsense (500) mutation and from microdissected regions of positive and negative staining from 1 (1298) of the 2 with positive and negative areas. The second of these (1094) had insufficient tissue in the FFPE block. Primers were designed to amplify short products containing the known variants found in each tumour, and the products were sequenced. Neither of the positively-staining cases contained the mutation found in the fresh-frozen sample (data not shown). In case 1298, with positive and negative staining, the mutation was detected in the negative part of the section but not the positive part, indicating genomic heterogeneity within this tumour (Fig. 4).

Of the six tumours with mutations that affect splicing, four showed loss of expression, one showed positive staining and one showed both positive and weakly staining areas. FFPE tissue was available for mutation analysis for the sample with positive staining (359), and this showed absence of the splicing mutation that had been detected in the related fresh-frozen sample. All five tumours with missense mutations showed positive staining, two of which (271 and 1338) also had areas of tissue with no expression. FFPE tissue from all of these was microdissected and examined for the presence of the missense mutation. In the two samples with positive and negative areas, both positive and negative areas were examined in one and only positive areas in the other. Mutation was detected in all samples analysed, indicating that none of these missense mutations affect protein stability. IHC data were not available for the three tumours (670, 945 and 991), which had mutations that directly or indirectly result in in-frame deletion.

Interestingly, three tumours in which no mutation was detected showed complete absence of expression and a further nine showed chimeric staining pattern with both positive and negative areas. In total, 14 of 120 tumours analysed by immunohistochemistry (12%) showed chimeric staining pattern, only 2 of which contained clearly inactivating mutations. In all of these cases, there was clear contiguity between positive and negative areas, with adjacent areas showing strong positivity or complete absence of staining. Tissue morphology was identical, and there was no indication that two separate tumours might be present and intermixing (Fig. 4).

**Figure 5.**
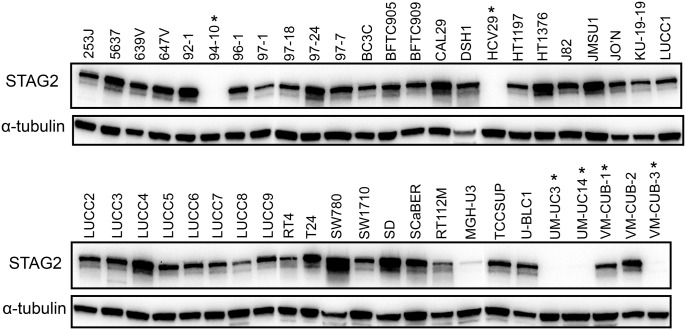
Immunoblot showing STAG2 expression in bladder tumour-derived cell lines. Proteins (30 µg) from 47 cell lines were analysed by western blot analysis using antibodies specific to STAG2. Asterisks denote cell lines carrying STAG2 mutations. Alpha-tubulin expression was used to assess protein loading.

We examined STAG2 expression in all 47 tumour cell lines by immunoblotting (Fig. 5). This confirmed the absence of expression in five lines with predicted inactivating mutations. The sixth line (VM-CUB-1) with a mutation that had been confirmed to affect splicing (Fig. 1; Supplementary Material, Table S4) showed protein expression. RT-PCR data showed that this resulted in in-frame deletion of exon 28 (35 amino acids). As this protein would not have a premature truncation, it may be stable and retain some function. MGH-U3, in which no mutation was detected, showed a very low level of STAG2 protein expression.

## DISCUSSION

We have identified a high frequency of inactivating mutation of *STAG2* in bladder cancer, with a significant association with low tumour grade and stage*.* Mutations were found in 32.6% of Ta tumours, and in 24.7% of bladder tumours overall. This represents the highest frequency of *STAG2* mutation detected in any cancer to date. A previous study reported absence of STAG2 protein expression in cell lines derived from a range of cancers (glioblastoma, Ewing's sarcoma, melanoma, haematologic, cervical and renal) and identified mutations or homozygous deletion in several of these. In tumours, mutations were identified in 4 of 68 glioblastomas (5.8%), a melanoma and a Ewing's sarcoma. The COSMIC database (25.7.13, date last accessed) lists only 57 confirmed somatic mutations in 7086 samples from a range of tumour types.

The high frequency of inactivating mutation in bladder cancer implies a cell type-specific effect of loss of function in the urothelium. In non-muscle-invasive tumours where mutation was most frequent, mutations in the promoter of telomerase (*TERT*) (>80%) (9,10) and activating mutations in *FGFR3* (∼70%) (11) are the most common events recorded to date. Mutations in *PIK3CA,* the catalytic subunit of phosphatidylinositol-3 kinase (PI3K), are found in 20–30% of cases (12). As expected, given the association of *STAG2* mutation with low tumour grade and stage, we also found significant association with these other mutational events in the non-invasive tumour group. The lower frequency of mutation or loss of expression found in bladder tumour cell lines is compatible with their origin from muscle-invasive bladder cancers in virtually all cases.

The vast majority of the 79 somatic mutations identified in tumour tissues were inactivating (nonsense, frameshift or splicing). Nonsense and frameshift mutations were distributed throughout the gene and regardless of position were associated with loss of protein expression in those cases where we were able to carry out immunohistochemistry, possibly caused by nonsense-mediated decay of the mRNA. A cluster of inactivating mutations (*n* = 10) was located in exon 31 between codons 1021 and 1065 (Table 1) the significance of which is not known. Phosphorylation sites have been identified at residues S1058, S1064 and S1065 and in the region C-terminal to this cluster (13). The C-terminal region also harbours putative nuclear localization signals (14). None of the missense mutations identified here affected these residues or any residues within the three LXXL motifs that show homology with transcriptional co-activators of the p160 family, one of which is implicated in ability of STAG2 to co-activate specific transcription factors (15).

The significance of the missense mutations identified is not clear. STAG2 protein sequence is highly conserved, and all residues affected by mutation are identical in vertebrates. This high level of conservation could indicate possible functional consequences of these alterations, although in several cases the changes were conservative. Here we tested for associations with grade, stage, gender and other mutations both with and without these mutations included, owing to this uncertainty. In no case did inclusion or exclusion of these affect the significance of the associations found. As this is a large protein and the missense mutations identified here show no clustering in specific regions of the protein or affect residues with known function, it is possible that they represent passenger mutations. Of the 57 somatic mutations recorded in the COSMIC database, 65% are missense and only 19% nonsense. We were able to assess protein expression in 5 of the 10 tumours with missense mutation. As all retained expression, we conclude that they do not affect protein stability. Functional studies will be required to assess their significance fully.

Overall we identified 3 tumours with complete absence of expression and 12 with chimeric staining that had no mutation identified. HRM is restricted to the detection of variants that can be amplified by PCR. Thus, large genomic deletions, such as that reported in U138MG by Solomon *et al*. (1), would not be detected by this technique, nor would smaller deletions where one or both of the deletion breakpoints are outside the region covered by the PCR. Furthermore, HRM, like any mutation scanning technique, has a mutation detection sensitivity of <100% (16). In a study that used single-strand conformation polymorphism analysis to search for mutations in colorectal, gastric, breast, non-small cell lung and prostate carcinomas (17), no mutations were identified but significant numbers of gastric, colorectal and prostate cancer samples showed absence of protein expression by immunohistochemistry. Further work is needed to assess other potential mechanisms of loss of expression. As *STAG2* has a CpG island in its promoter region, promoter hypermethylation is one possibility.

The frequency of chimeric expression pattern found in bladder tumours suggests that intra-tumour genomic evolution commonly involves loss of STAG2, even in low-grade, low-stage tumours, which are generally considered to represent an evolutionarily stable sub-group. We were able to confirm this genomic heterogeneity in one case where a known mutation could be detected. As the gene is large, it was not feasible to test for mutation in small microdissected tumour areas with lack of expression when no mutation had been found in the gross sample. Whole-genome amplification from such samples may allow clarification of the status of such tumours in future.

The finding of a higher frequency of mutation in females requires replication in an independent study. To date no other molecular features have been shown to differ between the bladder tumours of men and women. UC incidence is higher in males, but women tend to present later and their outcomes are less favourable (18), making gender an important factor in clinical decision making for UC management. The basis for the gender-related difference in incidence has not been precisely ascertained. Expression of the androgen receptor has been suggested to play a role not only in the development of the higher incidence of bladder cancer in males but also in determination of prognosis, although some conflicting results have been reported [reviewed in (19)]. It will be of interest to examine *STAG2* mutation in relation to both gender and the status of androgen receptor and its targets.

Our data indicate that loss of STAG2-cohesin function in mediating chromatid cohesion is unlikely to be the major effect of mutational inactivation in bladder cancer. Whole chromosome copy number alterations measured by array CGH showed an inverse relationship to *STAG2* mutation. As is widely reported, these were more common in tumours of high grade and stage where *STAG2* mutation was less common*.* Cohesin is also implicated in DNA repair where it mediates chromatid cohesion to allow repair of DNA double-strand breaks by homologous recombination. Again, defects in this process are predicted to lead to genomic instability, and our data showed an inverse relationship to sub-chromosomal copy number alterations, which could implicate this function. Thus, we hypothesize that the loss of STAG2 in the context of non-invasive bladder cancer has major impact on a cell-cycle and DNA repair-independent function.

Apart from the distinct roles of STAG1 and STAG2 in mediating telomere and centromere cohesion, respectively, only very recently has there been any information on functions of STAG1 and STAG2 that are either specific or redundant. Although STAG2 is more abundant in cells than STAG1, unique functions have not yet been reported. Some specific roles for STAG1 have been described following generation of STAG1 knockout mice, and results indicate that STAG2 cannot fulfil all functions of STAG1 (3,20). Results in STAG1-null cells indicate that STAG2-cohesin lacks ability to accumulate at promoters and shows reduced overlap with overall CTCF distribution compared with STAG1-cohesin. The development of STAG2 knockout mice may clarify which, if any, of the many functions of cohesin specifically involve STAG2. These functions have been reviewed recently [(21,22)].

In the recent study of STAG2 in glioblastoma, a clear effect of *STAG2* loss of function on the generation of aneuploidy was found (1). In this tumour type, transcriptional analysis of paired cell lines with and without STAG2 expression revealed no expression differences. In bladder, where no cohesin-related effect appears to result from loss of STAG2, an effect on transcription must be considered as an alternative possibility and now merits examination.

## MATERIALS AND METHODS

### Patient samples and DNA isolation

The study was approved by the Leeds-East Research Ethics Committee (99/156), and written informed consent was obtained from all patients. Cold cup biopsies of UC were collected, snap-frozen and stored in liquid nitrogen. The remainder of the tissue was embedded in paraffin for diagnostic assessment. The tumour panel consisted of 307 samples. Two hundred and ninety-seven were transitional cell carcinoma, eight were squamous cell carcinoma and two were small cell carcinoma. The transitional cell tumours comprised 4 pTaG1, 109 pTaG2, 25 pTaG3, 14 pT1G2, 62 pT1G3, 3 pT2G2, 67 ≥pT2G3 and 11 G2 and two G3 tumours with no underlying stroma (pTx) (23,24) (Supplementary Material, Table S1). Clinical data were collected from 232 patients. DNA was extracted from frozen sections containing ≥70% tumour cells and venous blood samples as described previously (12) and from FFPE tissue sections using a QIAamp DNA FFPE Tissue Kit (Qiagen).

### Urothelial cell lines

Forty-seven UC cell lines (253J, 5637, 639V, 647V, 92-1, 94-10, 96-1, 97-1, 97-18, 97-24, 97-7, BC3C, BFTC905, BFTC909, CAL29, DSH1, HCV29, HT1197, HT1376, J82, JMSU1, JO'N, KU-19-19, LUCC1, LUCC2, LUCC3, LUCC4, LUCC5, LUCC6, LUCC7, LUCC8, LUCC9, MGH-U3, RT112, RT4, SCaBER, SD, SW780, SW1710, T24, TCCSUP, U-BLC1, UM-UC3, UC-UC14, VM-CUB-1, VM-CUB-2 and VM-CUB-3) were investigated (Supplementary Material, Table S2). Telomerase-immortalized normal human urothelial cells (NHUC) (25) were used as controls. Cell line identity was verified by short tandem repeat DNA typing using Powerplex 16 kit (Promega). Profiles were compared with publically available data (ATCC and DSMZ), to matched patient samples (for LUCC1-LUCC9) or, where no reference profile was available, were confirmed as unique. DNA was extracted as described previously (12).

### STAG2 mutation analysis

Whole-genome amplification was carried out using the REPLI-g Mini kit (Qiagen) on DNA extracted from fresh-frozen tumour tissue samples. The entire coding sequence of *STAG2* and flanking intronic sequence was screened for variants using HRM analysis followed by bidirectional sequencing (16). Primers (Supplementary Material, Table S3) were designed using Primer3; http://frodo.wi.mit.edu/primer3/input.htm. The predicted melting profile of the PCR products was determined using DHPLCMelt (http://insertion.stanford.edu/melt.html), and in some cases, primers were redesigned or had short GC clamps added to make melting profiles as simple as possible.

PCR for HRM was carried out in 10 µl reactions overlaid with 20 µl of mineral oil. Each reaction contained 1× Acqua MasterMix (www.acquascience.com), 1× LCGreen+ (www.acquascience.com), 4 pmol of each primer and 1.5 µl of WGA DNA diluted 1/200 with TE. Cycle parameters were 95 °C for 15 min and then 45 cycles of 95 °C for 10 s, annealing for 15 s (59–61°C depending on primer pair) and 72 °C for 15 s; after a final denaturation at 94 °C for 30 s, reactions were cooled to 25 °C at 0.1°C/s to promote heteroduplex formation. HRM (16) was carried out using a HR96 LightScanner (www.acquascience.com). All samples with melting profiles that differed from wild-type samples were bi-directionally sequenced.

Tumour samples from male patients in which no mutation was identified on initial screening were re-screened after spiking with DNA from a male wild-type control sample to a concentration of ∼20% of amplifiable templates in the spiked sample. Cell line DNA samples were all screened after spiking with 15% of wild-type control DNA from a female sample.

PCR from FFPE sections was carried out in 10 µl reactions containing 1× HotStarTaq mastermix (Qiagen), 4 pmol of each primer and 1 µl of FFPE DNA. Cycle parameters were 95 °C for 15 min and then 40 cycles of 95 °C for 30 s, annealing for 30 s (50–56°C depending on primer pair) and 72 °C for 30 s followed by 72 °C for 10 min.

Sequencing was performed using a Big Dye Terminator Ready Reaction Mix v1.1 kit and primers described in Supplementary Material, Table S3. Data were collected using an Applied Biosystems 3130xl Genetic Analyser. Data were analysed by visual inspection of electropherograms and using Mutation Surveyor software (SoftGenetics, Inc.).

For 78 of 79 tumour samples containing variants, the variant was confirmed in a second PCR using DNA that had not been whole-genome-amplified. One variant (tumour 1135) was confirmed in a second PCR from whole-genome-amplified DNA. Somatic mutation status in tumours was confirmed by analysis of normal matched blood samples except for samples 271, 595, 991, 1294, 1415 and 1434.

### RNA isolation, cDNA synthesis and RT-PCR

Total RNA was extracted from frozen sections containing at least 70% tumour cells using a PicoPure RNA Isolation Kit (Nikon UK Limited). cDNA was synthesized using 250–500 ng of total RNA and Superscript II (Invitrogen) according to the manufacturer's instructions. PCR was performed in a volume of 25 µl containing 1 × PCR buffer, 1.5 mm MgCl_2_, 0.2 mm dNTPs, 0.2 µm of each primer (see Supplementary Material, Table S4), 2.5 units GoTaq DNA polymerase (Promega) and 5 µl of cDNA (diluted 1 in 5 prior to use). Cycle parameters were 95°C for 5 min and then 45 cycles of 95°C for 30 s, 55°C for 30 s and 72°C for 30 s followed by 72°C for 10 min.

### Immunohistochemistry

Tumour tissue sections were deparaffinized in xylene, rehydrated and endogenous peroxidase activity blocked in 3% hydrogen peroxide. Sections were boiled in 10 mm citric acid buffer (pH6) for 2 min, non-specific binding was blocked in 1× casein solution (Vector Laboratories) for 20 min, followed by incubation with primary antibody (1:200; sc-81852; Santa Cruz) for 1 h at room temperature, detection using the X-Cell Plus Polymer HRP detection Kit (Menarini Diagnostics), visualization with 3,3′ diamino-benzidine terahydrochloride (DAB; Vector Laboratories) and counterstaining with haematoxylin. All runs included a no primary antibody control.

### Protein extraction and western blot analysis

Cells were cultured to 70–80% confluence and then lysed in RIPAE buffer (1% Triton X-100, 1 mm EDTA, 0.5% sodium deoxycholate, 0.1% sodium dodecyl sulphate in phosphate-buffered saline) containing protease inhibitors (P8340; Sigma) and phosphatase inhibitors (P5726; Sigma). Proteins (30 µg) were separated under denaturing conditions in 7.5% SDS polyacrylamide gels and then transferred to Hybond-C super membrane (GE Healthcare Life Sciences). Blots were incubated with primary antibodies specific to STAG2 (1:1000; sc-81852; Santa Cruz) overnight at 4°C. Horseradish peroxidase-conjugated secondary antibodies and a Luminata Forte Western HRP substrate (Millipore) were used for chemiluminescent detection of bound antibody. Blots were incubated in stripping buffer (50 mm Tris pH7.5, 10 m urea) for 1 h at 55°C and reprobed with α-tubulin antibodies (1:3000; MCA77G; AbD Serotec) for the assessment of protein loading.

### Statistics

A Fisher's exact test was used to test for association between *STAG2* mutation status and tumour grade, patient gender and the mutation status of *FGFR3*, *PIK3CA* and RAS [data from (7) and unpublished data]. Chi-square analysis was used to test for association with tumour stage. Fraction of genome altered was as previously defined (7). *t*-tests were used to test for significant differences in FGA between *STAG2* mutant and wild-type samples. A Mann–Whitney *U*-test was conducted to test for significant differences in whole chromosome copy number alterations between *STAG*2 mutant and wild-type samples. Log-rank tests were carried out to test for the association between *STAG2* mutation status and time to recurrence. As the functional significance of missense mutations was not clear and it was considered that they may represent passenger events, all tests were carried out twice, with these samples designated either mutant or wild type.

## SUPPLEMENTARY MATERIAL

Supplementary Material is available at *HMG* online.

## FUNDING

This work was supported by grants from Cancer Research UK (C6228/A5433; C6228/A12512; C37059/A11941). Funding to pay the Open Access publication charges for this article was provided by Cancer Research UK (C37059/A11941).

## Supplementary Material

Supplementary Data
