# Supplementary material for: Frequent inactivating mutations of STAG2 in bladder cancer are associated with low tumour grade and stage and inversely related to chromosomal copy number changes
Source: Hum Mol Genet. 2013 Nov 22;23(8):1964–74. doi: 10.1093/hmg/ddt589 (PMC3959811; doi:10.1093/hmg/ddt589)
Supplement: Supplementary Data [file supp_23_8_1964__index.html]

Frequent inactivating mutations of STAG2 in bladder cancer are associated with low tumor grade and stage and inversely related to chromosomal copy number changes — Frequent inactivating mutations of STAG2 in bladder cancer are associated with low tumour grade and stage and inversely related to chromosomal copy number changes — Frequent inactivating mutations of STAG2 in bladder cancer are associated with low tumour grade and stage and inversely related to chromosomal copy number changes — Supplementary Data 

# Frequent inactivating mutations of *STAG2* in bladder cancer are associated with low tumour grade and stage and inversely related to chromosomal copy number changes

## Supplementary Data

Supplementary Data

**Files in this Data Supplement:**

- Supplementary Figure 1 - pdf file
- Supplementary Table 1 - pdf file
- Supplementary Table 4 - pdf file
- Supplementary Table 5 - pdf file
- Supplementary Data - Docx file
- Supplementary Table 2 - docx file
- Supplementary Table 3 - docx file
