# Supplementary figures and images for: Frequent inactivating mutations of STAG2 in bladder cancer are associated with low tumour grade and stage and inversely related to chromosomal copy number changes
Source: Hum Mol Genet. 2013 Nov 22;23(8):1964–74. doi: 10.1093/hmg/ddt589 (PMC3959811; doi:10.1093/hmg/ddt589)

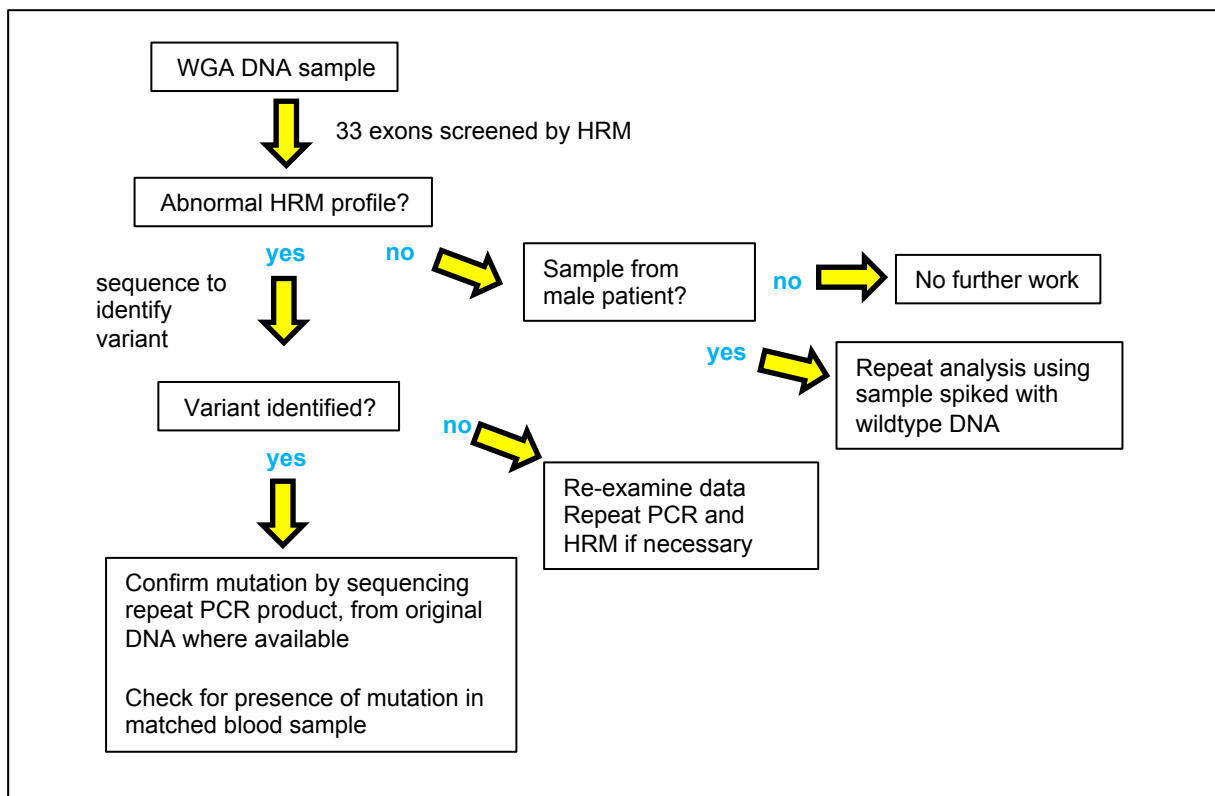

**Supplementary Figure 1.**

Supplement: Supplementary Data [file supp_ddt589_ddt589supp_fig1.pdf]
