# Supplementary material for: Frequent inactivating mutations of STAG2 in bladder cancer are associated with low tumour grade and stage and inversely related to chromosomal copy number changes
Source: Hum Mol Genet. 2013 Nov 22;23(8):1964–74. doi: 10.1093/hmg/ddt589 (PMC3959811; doi:10.1093/hmg/ddt589)
Supplement: Supplementary Data [file supp_ddt589_ddt589supp_table1.pdf]

Supplementary Table 1.

Sample	Gender	Tumour Stage	Tumour Grade	Tumour Pathology	Time to recurrence (months)	Follow up time (months) - see footnote	% clones altered in aCGH	Number of whole chromosome alterations detected by aCGH	Genomic position (GRCh37) of variation	Position of variant in transcript NM_001042749 (numbered with A of ATGi as nucleotide 1)	Predicted effect of variant at primary amino acid sequence level	Effect (Splice mutations supported by RNA evidence unless indicated otherwise)	IHC	FGFR3	PIK3CA	RAS	TP53
45	M	Ta	G2	TCC	26	40	9.50	1					ND	p.S249C	WT	WT	
94	M	T1	G3	TCC	3	17	12.77	1					+ & -	p.R248C	WT	WT	WT
95	M	≥T2	G2	TCC			14.67	1					ND	WT	WT	WT	WT
110	M	Ta	G3	TCC	23	153							ND	WT	WT	WT	
113	M	Ta	G3	TCC	12	18	13.49	4					+	WT	WT	WT	WT
137	M	≥T2	G3	TCC	33	126	64.50	3	X:123220428 C>T	c.3085 C>T	p.Q1029*	Nonsense	ND	WT	WT	WT	p.E271K
140	F	T1	G3	TCC	6	127	14.04	3	X:123220417 delT	c.3074 delT	p.F1025fs	Frameshift	ND	WT	p.E542K	WT	WT
169	F	≥T2	G3	TCC	No recurrence	20							ND				
228	M	T1	G3	TCC	5	47	19.60	2					+	p.S249C	WT	WT	WT
239	M	Ta	G1	TCC	No recurrence	121	0.00	0					+	WT	WT	KRAS p.G12R	WT
243	M	T1	G3	TCC	No recurrence	7	14.61	3					+	WT	WT	WT	WT
248	F	Ta	G2	TCC	No recurrence	66							ND				
264	M	Ta	G2	TCC	8	118	1.82	0					ND	WT	p.E545K	KRAS p.G12V	WT
271	M	T1	G3	TCC			20.45	4	X:123200222 T>C	c.2201 T>C	p.L734P	Missense	+ & -	WT	WT	WT	p.K132N
277	M	T1	G2	TCC	6	61							ND				
282	M	Ta	G2	TCC	39	84							ND	p.S249C	WT	WT	WT
287	M	Ta	G2	TCC	9	118	17.23	0					+	p.S249C	WT	WT	WT
291	F	≥T2	G3	TCC	14	18	22.85	2					+	WT	WT	WT	WT
299	F	Ta	G2	TCC	No recurrence	44	8.24	2					ND	p.S249C	WT	WT	WT
305	M	T1	G3	TCC									ND				
312	M	Ta	G2	TCC	17	92	10.00	2					+	p.S249C	WT	WT	WT
315	M	T1	G3	TCC	No recurrence	116	16.86	1	X:123179050 C>T	c.499 C>T	p.Q167*	Nonsense	ND	WT	p.D1017H	WT	p.R213W or p.R213X
322	F	Ta	G1	TCC	24	47			X:123191812 delinsTG	c.1401 delinsTG	p.F468fs	Frameshift	ND	p.S249C	WT	WT	
329	M	≥T2	G3	TCC	No recurrence	21							ND				
339	M	Ta	G2	TCC	5	25	8.39	2	X:123200233 C>T	c.2212 C>T	p.H738Y	Missense	ND	p.Y375C	WT	WT	WT
344	F	T1	G3	TCC	No recurrence	104	20.34	3					ND	p.S249C	WT	WT	WT
355	F	≥T2	G3	TCC	14	15	51.15	3					+	WT	WT	WT	p.R280T
356	F	Ta	G2	TCC	60	67							ND	p.S249C	p.E542K	WT	
358	F	Ta	G2	TCC	No recurrence	111	14.76	0					-	WT	WT	WT	WT
359	F	Ta	G2	TCC	5	111	0.12	0	X:123171474 G>T	c.385+1 G>T	p.?	Splice (presumed)	+	p.S249C	WT	WT	WT
360	M	≥T2	G3	TCC			26.92	3					+	WT	WT	WT	WT
364	F	T1	G3	TCC			25.88	2					-	WT	p.E545K	KRAS p.G12S	p.E198X
366	F	Tx	G2	TCC			17.03	0					+	WT	WT	WT	WT
374	F	T1	G3	TCC			30.49	0					ND	WT	WT	WT	WT
385	F	T1	G2	TCC	8	107	7.38	0	X:123181275 C>T	c.739 C>T	p.Q247*	Nonsense	-	p.S249C	WT	WT	WT
393	M	T1	G3	TCC	11	63							ND	WT	WT	HRAS p.G12S	
397	M	Ta	G2	TCC	No recurrence	106	0.00	0	X:123220496_123220497 dupTG	c.3153_3154 dupTG	p.G1052fs	Frameshift	-	p.S249C	p.E542K; p.E545K	KRAS p.G12D	WT
406	F	Ta	G2	TCC	No recurrence	106	3.69	0					ND	p.S249C	WT	WT	WT
411	M	T1	G3	TCC			71.61	2					ND	WT	WT	WT	WT
417	F	≥T2	G3	TCC			23.17	1	X:123197742_123197760 del19	c.1866_1884 del19	p.K622fs	Frameshift	-	WT	p.E542K	KRAS p.G12V	WT
418	F	T1	G3	TCC	No recurrence	105	7.55	2					ND	WT	p.E545K	WT	WT
428	M	Tx	G2	TCC	No recurrence	131							ND	WT	p.E545K	WT	WT
433	M	≥T2	G3	TCC	9	11	19.42	2					+	p.S249C; p.Y375C	WT	WT	WT
438	F	≥T2	G3	TCC	No recurrence	14	24.29	3					+	p.Y375C	WT	WT	p.H214R; p.R280K
443	F	Ta	G2	TCC	No recurrence	9							ND	p.S249C	WT	WT	
445	F	Ta	G3	TCC	17	17							ND	p.S249C	WT	WT	
451	M	Ta	G2	TCC	No recurrence	53							ND				
454	M	Ta	G2	TCC	14	101	0.35	0	X:123195666_123195667 dupGT	c.1580_1581 dupGT	p.T528fs	Frameshift	+	p.G372C	p.E542K	WT	WT
457	M	Ta	G2	TCC			12.97	2					+	p.S249C	p.M1043I	WT	WT
461	M	T1	G3	TCC	8	104	9.19	2					+	p.S249C	WT	WT	WT
466	M	≥T2	G3	TCC									ND				
468	M	T1	G2	TCC	36	104	5.30	1					+	WT	WT	WT	WT
469	F	Ta	G2	TCC									ND	WT	WT	NRAS p.Q61R	WT
482	F	Ta	G3	TCC	No recurrence	103	24.50	4					ND	p.S249C	WT	WT	WT
487	M	Ta	G2	TCC									ND				
489	F	Ta	G3	TCC	No recurrence	103	0.00	0					+	WT	p.E545K	WT	p.E180K
491	F	>T2	G3	SCC									ND	WT	WT	WT	p.R273L; p.Q331X
494	F	Ta	G2	TCC	No recurrence	103	0.32	0	X:123191758_123191762 del5	c.1347_1351 del5	p.R451fs	Frameshift	-	p.S249C	WT	WT	WT
498	F	≥T2	G3	TCC	No recurrence	20	16.08	1					ND	WT	WT	WT	WT
500	F	≥T2	G3	TCC			12.37	3	X:123220382 C>T; X:123220428 C>T	c.3054-15 C>T; c.3085 C>T	p.(=); p.Q1029*	No effect; Nonsense	+	p.S249C	WT	WT	WT
504	F	≥T2	G3	TCC			31.47	3					+	WT	WT	HRAS p.G12R	WT
511	M	Ta	G3	TCC	5	103	18.07	1	X:123220440 C>T	c.3097 C>T	p.R1033*	Nonsense	-	p.S249C	WT	WT	WT
513	M	≥T2	G2	TCC	No recurrence	103	29.69	1					ND	WT	p.E542K	WT	WT
518	M	Ta	G2	TCC	No recurrence	102	0.00	0					+	p.K652E	p.E542K	WT	WT
519	M	T1	G3	TCC	No recurrence	8	32.54	1					ND	p.R248C	WT	WT	p.E298X
525	M	T1	G3	TCC					X:123197834 C>G	c.1958 C>G	p.S653*	Nonsense	ND				
529	M	Tx	G2	TCC			14.52	1	X:123224554_123224557 dup4	c.3407_3410 dup4	p.S1137fs	Frameshift	ND	p.S249C	WT	WT	WT
535	F	Ta	G2	TCC	8	58							ND	p.S249C	WT	WT	
536	M	Ta	G2	TCC	15	102	0.00	0	X:123200250 G>A	c.2229 G>A	p.W743*	Nonsense	-	p.S249C	p.E545K	WT	WT
540	M	Ta	G2	TCC	5	102	15.01	2					+ & -	p.S249C	WT	WT	WT
554	M	T1	G2	TCC			9.25	3					+	p.S249C	WT	WT	WT
559	M	≥T2	G3	TCC	No recurrence	78							ND				
561	M	T1	G3	TCC	No recurrence	16	11.30	1					+	WT	p.H1047R	HRAS p.G13V	WT
567	M	T1	G3	TCC	50	61	31.01	3					+	WT	WT	WT	WT
571	F	T1	G3	TCC	6	36	10.69	3					+	p.S249C	WT	WT	WT

572	M	T1	G3	TCC	No recurrence	100	19.08	3					+	p.S249C	WT	WT	p.R249S
575	F	Ta	G2	TCC	No recurrence	100	2.25	0					-	p.R248C	p.E545K	WT	WT
577	F	≥T2	G3	TCC	No recurrence	14	24.50	1					+	WT	WT	WT	p.E180K
578	M	Ta	G2	TCC	10	99	0.98	0	X:123224422_123224443 del22	c.3278-3_3296 del22	p.?	Splice	-	p.Y375C	WT	WT	WT
579	F	T1	G2	TCC	No recurrence	99	9.19	1					+	p.S249C	p.E545K	WT	WT
582	F	≥T2	Small cell carcinoma	Small cell carcinoma					X:123185066 C>G	c.1113 C>G	p.F371L	Missense	ND	WT	WT	WT	
584	M	Ta	G3	TCC	No recurrence	99	0.00	0					ND	p.S249C	WT	WT	WT
588	F	≥T2	G3	TCC	No recurrence	99	43.50	2					+	WT	WT	WT	Splicing error (c.375+5 G>A)
589	M	T1	G3	TCC	No recurrence	99	16.48	1					+	WT	WT	WT	WT
593	M	T1	G2	TCC	6	99	12.36	2					+	p.S249C	WT	WT	WT
594	M	≥T2	G3	TCC	No recurrence	21	63.54	4					ND	WT	WT	WT	WT
595	M	≥T2	G3	TCC			71.06	6	X:123159695 C>G	c.50 C>G	p.S17*	Nonsense	ND	WT	WT	WT	p.S90fs
605	F	Ta	G2	TCC	No recurrence	124							ND	WT	p.E542K	KRAS p.Q61H	
610	F	≥T2	G3	TCC									ND	WT	WT	WT	
620	M	Ta	G2	TCC	No recurrence	23	5.49	1					ND	p.S249C	WT	WT	
621	M	T1	G3	TCC	36	65							ND	WT	WT	WT	
623	M	≥T2	G3	TCC									ND				
636	M	Ta	G3	TCC	No recurrence	97	21.04	2					ND	p.Y375C	p.E545K	WT	WT
643	F	Tx	G2	TCC	No recurrence	6	0.00	0	X:123220486 delT	c.3143 delT	p.L1048fs	Frameshift	-	p.G372C	p.E542K	WT	WT
647	M	Tx	G2	TCC	6	54							ND				
653	M	≥T2	G3	TCC			34.18	1					+	WT	WT	WT	p.M169X
657	F	Ta	G2	TCC	No recurrence	96	14.35	2					ND	WT	p.Q643R	WT	WT
658	F	Ta	G2	TCC	No recurrence	96	0.23	0					+	p.S249C	p.E542K	WT	WT
659	M	≥T2	G3	TCC	No recurrence	62							ND	WT	p.E545K	WT	
669	F	≥T2	G3	TCC									ND				
670	M	≥T2	G3	TCC					X:123211910 delT	c.2775+2 delT	p.?	Splice	ND				
672	F	Ta	G2	TCC	No recurrence	96	0.17	0					+	WT	WT	HRAS p.Q61R	WT
675	M	Ta	G2	TCC	No recurrence	96	7.35	2					ND	p.R248C	WT	WT	WT
676	M	Ta	G2	TCC	9	53							ND				
677	M	≥T2	G3	TCC	No recurrence	5	11.27	0					ND	WT	WT	WT	p.E51X
680	M	Ta	G2	TCC	11	33							ND	p.S373C	WT	WT	
685	M	≥T2	G3	TCC	No recurrence	49							ND				
695	F	Ta	G2	TCC	4	95	7.67	0					ND	WT	WT	WT	WT
701	M	≥T2	G3	TCC	No recurrence	14	4.83	0					ND	WT	WT	WT	p.R249S
712	M	Ta	G2	TCC	22	47							ND	p.S249C	p.M1043I	WT	WT
717	F	Ta	G2	TCC			15.85	2	X:123185024_123185025 delCA	c.1071_1072 delCA	p.N357fs	Frameshift	ND	p.S249C	WT	WT	WT
718	F	Ta	G2	TCC	No recurrence	29	23.34	5					+	p.S249C	WT	WT	WT
734	M	Ta	G3	TCC	11	17	36.08	3					+	WT	WT	WT	WT
736	M	Ta	G3	TCC	No recurrence	10	6.30	1	X:123200022 C>G	c.2097-3 C>G	p.?	Splice	ND	p.Y375C	WT	HRAS p.G12S	WT
741	F	≥T2	G3	TCC			58.50	6					+	WT	WT	WT	p.R273H
778	M	≥T2	G3	TCC	8	93	15.30	1					+	p.S373C	WT	WT	WT
787	M	T1	G2	TCC									ND	WT	WT	KRAS p.G12V	
798	M	T1	G3	TCC	No recurrence	92	50.32	5					ND	WT	WT	WT	p.Y163C
806	M	T1	G3	TCC			25.68	3					+	WT	WT	WT	WT
811	F	Ta	G2	TCC	10	92	0.03	0	X:123184997 dupT	c.1044 dupT	p.T349fs	Frameshift	ND	p.S249C	p.H1047R	WT	WT
818	M	≥T2	G3	TCC	No recurrence	8	27.81	1					+	WT	WT	WT	WT
826	M	≥T2	G3	TCC	No recurrence	12	14.76	1					+	WT	WT	WT	WT
836	F	Ta	G2	TCC	10	26							ND				
837	M	Ta	G1	TCC	No recurrence	91	2.80	0					+	p.S249C	WT	WT	WT
844	F	Ta	G2	TCC	No recurrence	91	3.95	1						p.S249C	p.E542K	WT	WT
848	M	T1	G3	TCC	No recurrence	6	55.18	5					+	WT	WT	WT	p.E221X
856	M	≥T2	G3	TCC									ND				
860	M	Tx	G2	TCC	34	91	4.87	1	X:123205173 G>A	c.2533 G>A	p.? & p.D845N	Splice and missense	ND	p.S249C	p.E545K	WT	WT
865	F	Ta	G2	TCC	10	110			X:123195101 dupG	c.1444 dupG	p.D482fs	Frameshift	ND	p.S249C	p.E545K	WT	
866	M	T1	G3	TCC	No recurrence	91	3.83	1					+ & -	p.K652N	WT	WT	WT
867	M	Ta	G3	TCC									ND				
868	M	Ta	G2	TCC	No recurrence	91	0.12	0	X:123181345 dupA	c.809 dupA	p.R271fs	Frameshift	ND	p.S249C	WT	WT	WT
869	F	T1	G2	TCC	No recurrence	47			X:123195677 delC	c.1591 delC	p.Q531fs	Frameshift	ND	p.S249C	p.E545K	KRAS p.G12A	
884	M	T1	G3	TCC	No recurrence	90	24.64	2					+	WT	WT	WT	p.R273H
893	M	≥T2	G3	TCC			43.44	1					ND	WT	WT	WT	p.C275Y
894	F	Ta	G1	TCC	27	90	0.09	0					ND	WT	WT	HRAS p.Q61L	WT
924	F	Ta	G2	TCC	No recurrence	34	6.20	0					+	WT	WT	HRAS p.Q61L	WT
928	M	≥T2	G3	TCC	No recurrence	13							ND				
929	F	T1	G3	TCC									ND				
930	M	T1	G3	TCC	5	89	68.73	8					+	WT	WT	WT	p.K139E
933	F	Ta	G2	TCC	11	13	1.27	0					+	p.S249C	p.E542K	WT	WT
934	F	Tx	G2	TCC	9	89	7.78	2					+ & -	p.Y375C	p.E545K	WT	WT
944	M	T1	G3	TCC	3	3	45.80	6					+	WT	WT	WT	p.R273C
945	F	Ta	G3	TCC	No recurrence	57			X:123200277_123200302 del26	c.2256_2265+16 del26	p.?	Splice	ND	p.Y375C	p.H1047R	WT	
946	F	Ta	G2	TCC	9	88	14.67	0					ND	WT	WT	WT	WT
949	M	≥T2	G3	TCC									ND				
957	M	≥T2	G3	TCC									ND				
960	F	Ta	G2	TCC	4	28							ND	p.Y375C	WT	WT	
961	M	Ta	G2	TCC	9	87	0.06	0	X:123199725 G>T	c.2026-1 G>T	p.?	Splice	-	p.S373C	WT	WT	WT
963	F	Ta	G2	TCC	24	87	1.53	0					+	p.S249C	WT	WT	WT
966	M	Ta	G2	TCC	No recurrence	87	21.21	3					+	WT	WT	WT	WT
968	M	≥T2	G3	TCC									ND				
969	M	T1	G3	TCC			8.52	0					+	p.Y375C	WT	WT	WT
979	M	T1	G3	TCC	5	86	21.09	4					+ & -	p.S249C	WT	WT	WT
980	F	Tx	G2	TCC	9	86	8.24	1					+ & -	p.S249C	WT	WT	WT
983	M	T1	G2	TCC	No recurrence	29	1.12	0	X:123200228_123200232 del5	c.2207_2211 del5	p.C736fs	Frameshift	-	p.Y375C	p.H1047R	WT	WT
985	M	Ta	G2	TCC	No recurrence	105		0	X:123181311 C>T	c.775 C>T	p.R259*	Nonsense	ND	p.G372C	p.H1047L	WT	

987	M	T1	G2	TCC					X:123195067 A>G	c.1417-7 A>G	p.?	Splice	ND	WT	WT	KRAS p.G12V	WT
989	M	Ta	G3	TCC	13	84	7.80	0	X:123215342 A>G	c.2888 A>G	p.Q963R	Missense	+	WT	WT	WT	WT
990	F	Ta	G2	TCC	5	98	0.00	0	X:123200229_123200240 del12	c.2208_2219 del12	p.C736*	Nonsense	ND	p.Y375C	p.E542K	WT	
991	F	Tx	G2	TCC	No recurrence	112	18.90	3	X:123179176_123179193 del18	c.625_642 del18	p.D209_A214del	In-frame deletion	ND	WT	p.E542K	KRAS p.G12C	
994	F	≥T2	G3	TCC	No recurrence	20							ND	WT	p.E542K	WT	
995	M	Tx	G2	TCC	24	91							ND	p.S249C	p.H1047R	WT	
996	F	Ta	G2	TCC	38	111							ND				
1005	M	Ta	G2	TCC	9	111							ND	p.S249C	p.E542K	WT	WT
1006	M	Ta	G2	TCC	6	30	7.87	0					ND	p.S249C	WT	WT	p.G108R
1009	M	Tx	G3	TCC	No recurrence	6	44.13	4					ND	WT	WT	WT	p.R175H
1010	M	T1	G3	TCC	No recurrence	84	6.53	0					+	WT	WT	HRAS p.R68P	WT
1014	F	≥T2	G1	SCC									ND	p.S249C	WT	WT	
1017	M	Ta	G2	TCC	72	102	1.20	0	X:123197045 G>T	c.1811 G>T	p.R604L	Missense	ND	p.S249C	WT	WT	
1021	M	T1	G3	TCC	No recurrence	83	14.50	1					ND	WT	p.E545K	WT	p.Q331X; p.D259V
1025	F	T1	G2	TCC	4	83	10.50	1	X:123179156 C>A	c.605 C>A	p.S202*	Nonsense	-	WT	WT	WT	WT
1028	M	T1	G3	TCC	10	82	7.54	2	X:123197049 A>C; X:123210303 C>T	c.1815A>C; c.2655 C>T	p.L605F; p.(=)	Missense; Synonymous	+	WT	WT	WT	WT
1030	M	Ta	G2	TCC	7	9	20.09	3					ND				WT
1033	M	T1	G3	TCC	4	22	24.34	2					+	WT	WT	WT	p.I195T
1038	M	Ta	G2	TCC	No recurrence	61	0.00	0					ND	p.S249C	p.E542K	WT	
1040	F	≥T2	G3	SCC									ND				
1043	M	Ta	G3	TCC	No recurrence	81	31.21	6					ND	p.S249C	WT	WT	WT
1044	M	Ta	G2	TCC	3	81	2.97	0					+	WT	WT	WT	WT
1046	M	Ta	G2	TCC	17	81	24.01	2	X:123176411 A>G	c.386-8 A>G	p.?	Splice (presumed)	+ & weak	p.S249C	WT	WT	WT
1049	F	≥T2	G3	TCC			42.92	4					ND	WT	WT	WT	p.Y205C
1052	F	T1	G3	TCC	7	12	12.77	2	X:123164943 G>T	c.256 G>T	p.E86*	Nonsense	-	p.Y375C	p.E545K	WT	WT
1053	F	Ta	G2	TCC	No recurrence	1	0.00	0	X:123202456 C>T	c.2308 C>T	p.Q770*	Nonsense	ND	p.Y375C	WT	WT	
1058	M	Ta	G2	TCC	No recurrence	99	0.00	0					ND	WT	WT	WT	
1063	M	Ta	G2	TCC	7	98	4.60	0	X:123195162 delT	c.1505 del T	p.L502fs	Frameshift	ND	p.S373C	WT	WT	
1066	M	≥T2	G3	SCC									ND				
1067	M	Ta	G2	TCC	9	105							ND	WT	WT	HRAS p.Q61R	WT
1072	M	Ta	G3	TCC	8	24	20.11	1	X:123195215 T>A	c.1534+24 T>A	p.=	No effect	+ & -	WT	WT	WT	WT
1073	M	Ta	G3	TCC	7	15	23.22	5	X:123215302 G>A	c.2848 G>A	p.E950K	Missense	ND	WT	WT	WT	WT
1079	F	Ta	G3	TCC	No recurrence	78	12.72	1	X:123210190 C>T	c.2542 C>T	p.Q848*	Nonsense	ND	p.R248C	p.E545K	WT	WT
1081	F	T1	G3	TCC	No recurrence	78	0.00	0	X:123224511 dupA	c.3364 dupA	p.T1122fs	Frameshift	ND	p.A393E	p.H1047R	WT	p.Y234H
1082	F	Ta	G3	TCC	No recurrence	78	17.72	3					+	WT	WT	WT	WT
1083	M	Ta	G2	TCC	No recurrence	4			X:123215276 C>G	c.2822 C>G	p.S941*	Nonsense	ND	p.S249C	WT	WT	WT
1088	M	Ta	G2	TCC	No recurrence	44							ND	WT	WT	WT	WT
1090	F	Ta	G2	TCC	4	39							ND	WT	WT	WT	WT
1091	M	Ta	G2	TCC	3	22	15.00	1					ND	p.S249C	p.E545K	WT	
1094	F	T1	G3	TCC	No recurrence	77	13.72	2	X:123227933 C>G	c.3644 C>G	p.S1215*	Nonsense	+ & -	p.S249C	WT	WT	WT
1096	M	Ta	G2	TCC	No recurrence	16	4.89	1					ND	p.Y375C	WT	WT	
1103	M	Ta	G3	TCC	No recurrence	76	0.23	0					+	WT	p.E542K; p.E545K	WT	WT
1109	M	≥T2	G3	TCC	51	60	21.32	0					+	WT	WT	KRAS p.G12D	WT
1111	M	Ta	G3	TCC	No recurrence	98			X:123217323 C>T; X:123220428 C>T	c.2977 C>T; c.3085 C>T	p.P993S; p.Q1029*	Missense; Nonsense	ND	p.Y375C	p.E545K	WT	
1116	F	Ta	G2	TCC	48	87	0.00	0	X:123191785 delC	c.1374 delC	p.N459fs	Frameshift	ND	p.S249C	WT	WT	
1117	M	Ta	G2	TCC	7	25	0.00	0					ND	WT	p.E542K	HRAS p.Q61R	
1118	M	T1	G3	TCC	12	75	12.31	1					+	p.R248C	p.E545K	WT	WT
1126	M	≥T2	G3	TCC	No recurrence	8	42.92	0					+	WT	WT	WT	p.E68D; p.A76V; p.R337C
1129	M	T1	G3	TCC	No recurrence	74	14.47	0					+	WT	WT	NRAS p.Q61L	WT
1130	M	Ta	G3	TCC			17.90	1					ND	p.S249C	p.E545K	WT	
1135	M	Ta	G3	TCC					X:123191812 delinsTG	c.1401 delinsTG	p.F468fs	Frameshift	ND	WT	p.E545K	WT	
1138	M	T1	G3	TCC	No recurrence	73	16.74	0					+	WT	WT	WT	WT
1142	M	Ta	G2	TCC	18	92	0.00	0	X:123210178_123210198 del21	c.2534-4_2550 del21	p.?	Splice	ND	p.S249C	p.E545K	WT	
1144	M	Ta	G2	TCC	No recurrence	92	0.00	0					ND	WT	WT	HRAS p.Q61R	
1145	M	T1	G3	TCC	No recurrence	73	28.74	1	X:123171455 C>T	c.367 C>T	p.Q123*	Nonsense	-	WT	WT	WT	WT
1148	F	≥T2	G3	TCC	No recurrence	24	24.68	5					+	WT	WT	WT	p.E271K; p.N310fs
1150	M	Ta	G3	TCC									ND				
1152	M	≥T2	G3	TCC									ND				
1153	M	T1	G3	TCC	3	19	8.75	1					+	p.R248C	WT	WT	Splicing error (c.672+1G>T)
1157	M	T1	G3	TCC			10.64	0					+	p.S249C	WT	WT	WT
1161	M	T1	G3	TCC	No recurrence	8	11.39	2					+	p.S249C	p.E545K	WT	WT
1165	F	≥T2	G3	SCC									ND				
1167	F	Ta	G2	TCC	No recurrence	84	0.00	0	X:123176440_123176447 del8	c.407_414 del8	p.F136fs	Frameshift	ND	p.G372C	WT	WT	
1172	F	≥T2	G3	TCC					X:123229288 A>G	c.3772 A>G	p.M1258V	Missense	ND				
1175	M	Ta	G2	TCC	16	16	11.40	1	X:123185248 A>G	c.1196+4 A>G	p.?	Splice	ND	p.G372C	WT	WT	
1176	F	≥T2	G3	TCC									ND				
1177	M	Ta	G2	TCC	3	90	0.00	0					ND	WT	WT	HRAS p.G12S	
1180	F	≥T2	G3	TCC									ND	WT	WT	WT	
1185	F	Tx	G2	TCC	34	35	0.00	0	X:123202427 delinsCT	c.2279 delinsCT	p.R760fs	Frameshift	ND	p.Y375C	p.H1047L	WT	
1202	M	≥T2	G3	SCC									ND	WT	WT	WT	WT
1207	M	≥T2	G3	SCC									ND				
1210	F	T1	G3	TCC	8	70	20.20	2	X:123200024 G>A	c.2097-1 G>A	p.?	Splice	-	WT	WT	WT	WT
1212	M	T1	G3	TCC	No recurrence	10	29.20	3					+	WT	WT	WT	p.T140-Q144Del; p.E224K; p.C238Y
1213	F	Ta	G2	TCC			4.90	1					ND	p.S249C	p.H1047L	WT	
1214	F	Ta	G2	TCC	4	89	0.30	0	X:123200023 A>G	c.2097-2 A>G	p.?	Splice (presumed)	ND	p.S249C	WT	WT	
1220	M	T1	G2	TCC			1.01	1					+	p.S249C	WT	WT	WT
1229	M	T1	G3	TCC	6	12	15.10	0					+	p.S249C	WT	WT	WT
1230	M	T1	G3	TCC	11	68	15.74	2					+	p.S249C	WT	WT	WT
1231	M	Ta	G2	TCC	No recurrence	24	2.00	0	X:123176495 G>C	c.462 G>C	p.?	Splice	ND	p.R248C	p.H1047R	WT	
1234	M	T1	G3	TCC	53	68	24.60	1					+	WT	WT	WT	p.M246I
1238	M	Ta	G2	TCC	No recurrence	87	0.00	0					ND	p.S249C	p.E545K	WT	
1240	M	T1	G3	TCC	No recurrence	5	10.07	2					ND	WT	WT	KRAS p.G12R	WT

1248	F	≥T2	G3	TCC	No recurrence	26	70.57	1					+	WT	WT	WT	p.E258G
1264	F	T1	G3	TCC	No recurrence	65	27.36	3					+	WT	WT	WT	WT
1267	M	≥T2	G3	TCC	16	65	16.05	3					+	p.S249C	p.E545K	WT	WT
1272	M	Ta	G2	TCC	7	84	3.00	0					ND	p.S249C	WT	WT	
1273	F	Ta	G2	TCC	12	84	0.00	0	X:123215379 G>A	c.2924+1 G>A	p.?	Splice	ND	p.S249C	WT	WT	
1281	F	≥T2	G3	TCC									ND				
1283	M	Ta	G2	TCC	16	83	5.70	1					ND	p.S249C	WT	WT	
1285	M	Ta	G2	TCC	No recurrence	76	3.20	1					ND	p.S249C	p.E545K	WT	
1294	M	Ta	G2	TCC	3	49	6.50	1	X:123220406 C>G	c.3063 C>G	p.Y1021*	Nonsense	ND	p.S249C	WT	WT	
1298	M	Ta	G3	TCC	No recurrence	63	3.51	0	X:123196806 G>T	c.1693 G>T	p.E565*	Nonsense	+ & -	p.S249C	p.E545K	WT	WT
1300	M	T1	G3	TCC	3	15	35.67	7					+	WT	p.E545K	WT	p.L130P; p.D228H
1301	M	≥T2	G3	TCC	No recurrence	2	38.41	4					+	WT	WT	WT	p.R273C
1305	M	Ta	G2	TCC	No recurrence	39	4.00	1					ND	p.S249C	WT	WT	
1307	M	Ta	G2	TCC	No recurrence	45	7.30	1					ND	p.S249C	WT	WT	
1308	F	Tx	G2	TCC	11	89	5.60	0					ND	p.K652E	p.E545K	WT	
1310	M	T1	G3	TCC	43	62	25.52	1					+	WT	WT	WT	p.E258X
1311	M	T1	G3	TCC	12	16	6.21	1					+	p.G372C	p.E545Q	WT	WT
1313	M	Ta	G2	TCC	4	27	8.60	1					ND	p.S249C	WT	WT	
1315	M	T1	G3	TCC	No recurrence	62	0.14	0					+ & -	p.S249C	p.E545K	WT	p.R280T
1317	M	≥T2	G3	TCC	No recurrence	6	2.99	0					+	WT	WT	WT	p.K139N
1320	M	T1	G3	TCC			50.81	6					+	WT	WT	WT	p.Y234H
1321	F	≥T2	G3	TCC	No recurrence	12	7.28	1					+	p.S249C	p.H1047R	WT	p.R280K
1322	M	≥T2	G3	TCC	No recurrence	5	25.03	0					+	WT	WT	WT	WT
1324	M	T1	G3	TCC	2	61	12.11	1	X:123224595 C>G	c.3448 C>G	p.Q1150E	Missense	+	p.S249C	WT	WT	p.S90F; p.Q192X; p.G244V; p.E388Q
1325	M	T1	G3	TCC			28.31	5					+ & -	p.S249C	WT	WT	p.Y234H
1326	M	Ta	G2	TCC	No recurrence	86	7.50	2					ND	p.S249C	WT	WT	
1332	M	Ta	G2	TCC	No recurrence	86	6.10	1	X:123182867 C>T	c.832 C>T	p.Q278*	Nonsense	ND	p.S249C	WT	WT	
1335	F	Ta	G2	TCC	22	86	8.20	1	X:123220533_123220534 delinsT	c.3190_3191 delinsT	p.S1065fs	Frameshift	ND	p.S249C	p.E542K	WT	
1338	F	≥T2	G3	TCC	No recurrence	10	29.37	2	X:123197864 A>G	c.1988 A>G	p.D663G	Missense	+ & -	p.Y375C	p.E545K	WT	p.Q192X
1342	M	T1	G2	TCC	8	59	16.11	2					+ & -	p.S249C	WT	WT	WT
1345	M	T1	G2	TCC	No recurrence	59	12.92	2	X:123195581_123195652 del72	c.1535-40_1566 del72	p.?	Splice	-	p.S249C	WT	WT	WT
1349	M	T1	G3	TCC	No recurrence	39	20.89	3					+	WT	p.E545K	KRAS p.G12V	WT
1350	F	T1	G3	TCC									ND				
1352	M	≥T2	G2	TCC	No recurrence	58	36.97	3					ND	WT	WT	WT	WT
1361	M	Ta	G2	TCC	34	76	0.50	0					ND	p.S249C	p.E545K	WT	
1366	M	≥T2	G3	TCC			47.12	4					+	WT	WT	WT	p.R248Q
1370	M	Ta	G2	TCC	No recurrence	82	17.20	3					ND	p.S249C	WT	WT	
1380	M	Ta	G2	TCC	9	18	31.40	4					ND	p.S249C	WT	WT	
1383	F	Ta	G2	TCC	5	40	8.30	2	X:123184160_123184181 delinsCATCTTAC	c.1017+1_1017+22 delinsCATCTTAC	p.?	Splice	ND	p.Y375C	p.E545K	WT	
1385	F	Ta	G2	TCC	No recurrence	75	0.00	0					ND	WT	WT	WT	
1387	F	Ta	G2	TCC	23	82	5.10	0					ND	p.R248C	WT	WT	
1413	M	≥T2	G3	TCC									ND				
1415	F	Ta	G2	TCC	No recurrence	59	0.00	0	X:123195635 C>T	c.1549 C>T	p.Q517*	Nonsense	ND	p.S249C	p.H1047L	WT	
1417	M	Ta	G2	TCC	32	72	0.00	0					ND	WT	p.E545K	HRAS p.G13R	
1427	F	≥T2	G3	TCC									ND				
1434	M	Ta	G2	TCC	No recurrence	12	0.70	0	X:123220476 C>T	c.3133 C>T	p.R1045*	Nonsense	ND	p.R248C	WT	WT	
1436	M	Ta	G2	TCC	No recurrence	76			X:123171455 C>T	c.367 C>T	p.Q123*	Nonsense	ND	p.S249C	WT	WT	
1439	M	Ta	G3	TCC	No recurrence	75							ND	WT	WT	WT	
1440	M	Ta	G2	TCC	No recurrence	61	0.00	0					ND	p.K652E	p.E542K	WT	
1444	M	Ta	G2	TCC	No recurrence	37	14.80	1					ND	p.G372C	WT	WT	
1453	M	Ta	G2	TCC	18	54	10.70	1					ND	p.Y375C	p.E542K	WT	
1465	M	Ta	G2	TCC	7	74	12.20	2	X:123197784 C>A	c.1908 C>A	p.Y636*	Nonsense	ND	p.S249C	WT	WT	
1467	M	Ta	G2	TCC	14	62	4.90	1					ND	p.S249C	WT	WT	
1473	F	Ta	G2	TCC	No recurrence	74	0.30	0	X:123185131 C>T	c.1117-34 C>T	p.(=)	No effect	ND	p.S249C	p.E545K	WT	
1474	F	Ta	G2	TCC	56	67	0.00	0	X:123184989 A>C; X:123211840_123211843 del4	c.1036 A>C; c.2707_2710 del4	p.K346Q; p.T903*	Missense; Nonsense	ND	p.S249C	WT	WT	
1487	M	≥T2	G3	TCC									ND				
1516	M	≥T2	Small cell carcinoma	Small cell carcinoma									ND				
1529	M	≥T2	G3	TCC					X:123199756 C>T	c.2056 C>T	p.Q686*	Nonsense	ND				
1552	F	≥T2	G3	SCC									ND				
1560	M	≥T2	G3	TCC									ND				
1576	F	≥T2	G3	TCC									ND				
1595	M	≥T2	G3	TCC									ND				
1598	M	≥T2	G3	TCC									ND				
1602	F	≥T2	G3	TCC									ND				
1628	F	≥T2	G3	TCC									ND				
1636	M	Ta	G2	TCC	15	58	20.60	4					ND	p.S249C	p.E542K	WT	
1673	M	≥T2	G3	TCC									ND				
1696	M	≥T2	G3	TCC					X:123191765_123191798 del34	c.1354_1387 del34	p.G452fs	Frameshift	ND				

Array CGH was carried out using BAC arrays as described in Hurst et al Clinical Cancer Research 18: 5865-5877, 2012
Fraction of genome altered was classified as follows: A=<1.0% clones; B=1.0-<10% clones; C=10-<30% clones; D=>30% clones altered
