# Supplementary material for: Frequent inactivating mutations of STAG2 in bladder cancer are associated with low tumour grade and stage and inversely related to chromosomal copy number changes
Source: Hum Mol Genet. 2013 Nov 22;23(8):1964–74. doi: 10.1093/hmg/ddt589 (PMC3959811; doi:10.1093/hmg/ddt589)
Supplement: Supplementary Data [file supp_ddt589_ddt589supp_table4.pdf]

Supplementary Table 4. RT-PCR analysis of splicing mutations.

Sample	Splice site affected by mutation	cDNA	Primers used in RT-PCR reactions	Experimentally determined effect (RT-PCR and sequencing of RT product)	RNA level description of mutation based on RT-PCR findings	Protein level description of mutation based on RT-PCR findings
359	exon 7 5' splice site	c.385+1 G>T	n/d	RNA not available. Mutation predicted to destroy splice site and could cause skipping of exon 7, use of local cryptic splice site or both.	Presumed change(s): r.(289_385 del 97) and/or r.(385_386 ins86).	Presumed change(s): p.(S97fs) and/or p.G129fs).
578	exon 32 3' splice site	c.3278-3_3296 del22	Fwd 5'-ggaaagtggttgagggcatg-3'<br>Rev 5'-tgccttgcttcctcttgttg-3'	No normal-sized product produced. One smaller band from use of a cryptic 3'splice site in exon 32 and one larger band from both use of the cryptic 3' splice site and retention of intron 32.	r.3278_3302 del25	p.T1093fs
670	exon 28 5' splice site	c.2775+2 del T	Fwd 5'-tgcagcattttgtaagctaattg-3'<br>Rev 5'-aagcaaaacgtcgagcaagt-3'	Skipping of exon 28.	r.2674_2775 del102	p.Y892_Q925del
736	exon 23 3' splice site	c.2097-3 C>G	Fwd 5'-gattttctgcaagagggtgaa-3'<br>Rev 5'-gcaagttgccaaaggattaca-3'	The mutation destroys the real exon 23 3' splice site and introduces a new cryptic 3' splice site located 2 nucleotides upstream of the real site.	r.2096_2097 ins ag	p.N699fs
860	final nucleotide of exon 26	c.2533 G>A	Fwd 5'-caacattacctgaccaacgtga-3'<br>Rev 5'-actggcttcatcctcttgct-3'	Skipping of exon 26 is major product. Some product retaining exon 26 with mutated nucleotide at c.2533 also detected, suggesting this mutation acts as both splice and missense.	r.2359_2533 del175 and r.2533 g>a	p.A787fs and p.D845N
945	exon 24 5' splice site	c.2256_2265+16 del 26	Fwd 5'-cattgaagaggatcactgctttt-3'<br>Rev 5'-acagtagtattcacgttggtcag-3'	Skipping of exon 24.	r.2185_2265 del81	p.I729_K755del
961	exon 22 3' splice site	c.2026-1 G>T	Fwd 5'-gacgattagaaaagcatttg-3'<br>Rev 5'-catgggcattatgaaaagca-3'	Use of a cryptic 3' splice site located in exon 22 which results in the deletion of nucleotides c.2026_2032. Compare with UM-UC14.	r.2026_2032del7	p. G676fs
987	close to exon 17 3' splice site	c.1417-7 A>G	Fwd 5'-tccaaatgccaaccttgttaaga-3'<br>Rev 5'-tcagccgcttgtctaatggt-3'	Use of a cryptic 3'splice site in intron 16 created by the mutation.	r.1416_1417 ins uuuuag	p.L473fs
1046	close to exon 8 3' splice site	c.386-8 A>G	n/d	RNA not available. Predict use of cryptic 3' splice site introduced by mutation.	Presumed change r.(385_386 ins auuucag)	Presumed change p.(G129fs)
1072	intron 17	c.1534+24 T>A	Fwd 5'-ggtccaaatgccaaccttgt-3'<br>Rev 5'-tttcctgtccctcttcccac-3'	No evidence for abnormal splicing.	No evidence for abnormal splicing.	No evidence for abnormal splicing.
1142	exon 27 3' splice site	c.2534-4_2550 del21	Fwd 5'-tcattgcagtctgagttgctc-3'<br>Rev 5'-agggtcttagcacactgaattt-3'	Skipping of exon 27.	r.2534_2673 del140	p.D845fs
1175	exon 14 5' splice site	c.1196+4 A>G	Fwd 5'-tcttactgctctacaagggct-3'<br>Rev 5'-gccggtgagctgaataaacc-3'	Uses cryptic 5' splice site located 4 nt downstream of real exon 14 5' splice site, resulting in the insertion of the first 4 nt of mutant intron 14 between exons 14 and 15.	r.1196_1197 ins guag	p.S400*
1210	exon 23 3' splice site	c.2097-1 G>A	Fwd 5'-gattttctgcaagagggtgaa-3'<br>Rev 5'-gcaagttgccaaaggattaca-3'	Skipping of exon 23.	r.2097_2184 del88	p.N699fs
1214	exon 23 3' splice site	c.2097-2 A>G	n/d	RNA not available. Predict use of cryptic splice site within exon 23 and/or skipping of exon 23 as in 94-10 which has the same mutation.	Presumed changes [r.(2097_2115 del19), r.(2097_2184 del 88)]	Presumed changes [p.(A700fs), p.(N699fs)]
1231	final nucleotide of exon 8	c.462 G>C	Fwd 5'-agtcggtggtagatgattgga-3'<br>Rev 5'-cagccatggtaagtggataatct-3'	Skipping of exon 8.	r.386_462 del77	p.V130*
1273	exon 29 5' splice site	c.2924+1 G>A	Fwd 5'-tgcagcattttgtaagctaattg-3'<br>Rev 5'-taaaggtggatggctctccc-3'	Skipping of exon 29.	r.2776_2924 del149	p.L926fs
1345	exon 18 3' splice site	c.1535-40_1566 del72	Fwd 5'-gatagcatgtgggactgtgc-3'<br>Rev 5'-tgtcctatcatccaactgtgtct-3'	No normal-sized product produced. Faint bands on agarose gel did not produce sequence and are probably background. The mutation deletes the exon 18 3' splice site and as there are no local cryptic 3' splice sites, it is possible that one or both of the flanking introns is retained, producing a product too large to amplify.	r.0	p.0
1383	exon 12 5' splice site	c.1017+1_1017+22 delins CATCTTAC	Fwd 5'-aaaatgattggaaaacgagcca-3'<br>Rev 5'-agcccttgtagagcagtaaga-3'	Use of cryptic 5' splice site in intron 12. Inserts nucleotides from intron 12 between exons 12 and 13.	r.1017_1018 ins65	p.Q340fs
94-10	exon 23 3' splice site	c.2097-2 A>G	Fwd 5'-gattttctgcaagagggtgaa-3'<br>Rev 5'-gcaagttgccaaaggattaca-3'	Major product uses a cryptic 3' splice site in exon 23. Minor product skips exon 23	r.2097_2115 del19 and r. 2097_2184 del 88	p.A700fs and p.N699fs
UM-UC14	exon 22 3' splice site	c.2026-1 G>T	Fwd 5'-gacgattagaaaagcatttg-3'<br>Rev 5'-catgggcattatgaaaagca-3'	Use of a cryptic 3' splice site located in exon 22 which results in the deletion of nucleotides c.2026_2032. Also has use of a cryptic 3' splice site located in intron 21 which results in the insertion of 71 nucleotides between exons 21 and 22.	r.2026_2032 del7 and r.2025_2026 ins71	Both abnormal RNA products result in p.G676fs
VM-CUB-1	exon 28 3' splice site	c.2674-6_2686 dup	Fwd 5'-tgcagcattttgtaagctaattg-3'<br>Rev 5'-aagcaaaacgtcgagcaagt-3'	Skipping of exon 28.	r.2674_2775 del102	p.Y892_Q925del
VM-CUB-3	exon 7 3' splice site	c.289-2 A>G	Fwd 5'-gtccaaaccgaatgaatggtc-3'<br>Rev 5'-gtctaaacatttctgctgtgaca-3'	Use of cryptic 3' splice site in exon 7.	r.289_298 del10	p.S97fs
