# Supplementary material for: Frequent inactivating mutations of STAG2 in bladder cancer are associated with low tumour grade and stage and inversely related to chromosomal copy number changes
Source: Hum Mol Genet. 2013 Nov 22;23(8):1964–74. doi: 10.1093/hmg/ddt589 (PMC3959811; doi:10.1093/hmg/ddt589)
Supplement: Supplementary Data [file supp_ddt589_ddt589supp_table5.pdf]

Supplementary Table 5. Variants identified in cell lines

Cell line	Gender	Genomic position (GRCh37) of variation	Position of variant in transcript NM_001042749 (numbered with A of ATGi as nucleotide 1)	Predicted effect of variant at primary amino acid sequence level	Effect (Splice mutations supported by RNA evidence	Protein expression detected by immuno-blotting	FGFR3	PIK3CA	RAS	TP53
UM-UC14	M	X:123156446 G>A; X:123199725 G>T	c.-32 G>A; c.2026-1 G>T	p.?; p.?	Unknown effect; Splice	-	p.S249C	WT	WT	p.R280T
VM-CUB-3	M	X:123171375 A>G	c.289-2 A>G	p.?	Splice	-	WT	p.E545K	WT	p.P278L
U-BLC1	F	X:123196872 A>C	c.1731+28 A>C	p.=	No effect	+	WT	WT	WT	p.H296Y; p.Q331*
HCV29	M	X:123196990 dupA	c.1756 dupA	p.T586fs	Frameshift	-	WT	WT	WT	WT
94-10	M	X:123200023 A>G	c.2097-2 A>G	p.?	Splice	-	p.S249C	p.V136I	WT	WT
VM-CUB-1	M	X:123211801_123211819 dup	c.2674-6_2686 dup	p.?	Splice	+	WT	p.E542K	WT	p.R175H
UM-UC3	M	X:123217293_123217294 delinsT	c.2947_2948 delinsT	p.K983*	Nonsense	-	WT	WT	KRAS p.G12C	p.F113C
92-1	F	X:123234427 C>G	c.3787 C>G	p.L1263V	Missense	+	WT	WT	WT	p.R158C; p.E224K; p.D228H; p.R280T; p.E294K
