# Supplementary material for: Frequent inactivating mutations of STAG2 in bladder cancer are associated with low tumour grade and stage and inversely related to chromosomal copy number changes
Source: Hum Mol Genet. 2013 Nov 22;23(8):1964–74. doi: 10.1093/hmg/ddt589 (PMC3959811; doi:10.1093/hmg/ddt589)
Supplement: Supplementary Data [file supp_ddt589_ddt589supp.docx]

**Supplementary text**

**STAG2 splicing mutations: description of effects at RNA level**

The locations of cryptic splice sites on the genomic DNA sequence are shown with pink (intronic part of cryptic site) and green (exonic part of cryptic site) highlights. Thus cryptic 3’ splice sites are nnnn and cryptic 5’ splice sites are nnnn. Coding/exonic nucleotides are shown in uppercase, with intronic nucleotides in lowercase.

For all samples except tumors 359, 1046, 1214 and 1345, the descriptions below are of our findings from sequencing RT-PCR products. No RNA sample was available for tumors 359, 1046 and 1214. No STAG2 RT-PCR product was obtained for tumor 1345. In every case for which RNA was available, RNA from normal human urothelial cells was used as a control and in every case, this produced a single band of the expected size for the normally spliced product and was shown to be of normal sequence.

**Tumor 359 (c.385+1 G>T)**

No RNA was available for this sample. The mutation is predicted to destroy the 5’ splice site of exon 7. The most likely effects on splicing are predicted to be either skipping of exon 7, use of a cryptic 5’ splice site in intron 7 or a combination of both effects.

DNA sequence with position of mutation highlighted

123171402 **CATACAAGCA TGACCGAGAT ATAGCACTTC TTGACCTTAT CAACTTTTTT ATTCAGTGTT** 123171461

123171462 **CAGGCTGTAA AG**gtaagata t[a](http://www.ensembl.org/Homo_sapiens/ZMenu/TextSequence?db=core;factorytype=Location;g=ENSG00000101972;r=X:123094062-123556514;v=rs188012323;vf=48869732)ttt[a](http://www.ensembl.org/Homo_sapiens/ZMenu/TextSequence?db=core;factorytype=Location;g=ENSG00000101972;r=X:123094062-123556514;v=TMP_ESP_X_123171487;vf=57080215)tttt gaaatcagca gctctcaaat agtcacttcg 123171521

123171522 ttgttccacc taaagagatg ttgttagcac tttacatggt aaataacgct aaggcattat 123171581

Skipping of exon 7 would result in the final nucleotide of exon 6, c.288, being joined to the first nucleotide of exon 8, c.386: r.(289_385 del97). Exon 7 is 97 nucleotides in length, so the exon-skipped product would be out of frame: p.(S97fs).

Exon 6 Exon 8

GCUAUGCAGGAGUUGUCACAGCAGAAAUGUUUAGACAUAUGCAGAACUCUGAGAUAA

-A--M--Q--E--L--S--Q--Q--K--C--L--D--I--C--R--T--L--R--*

Use of the cryptic 5’ splice site in intron 7 would result in the inclusion of 86 bp of intron sequence between exon 7 and exon 8: r.(385_386 ins86). At the protein level, this would result in p.(G129fs).

Exon 7 mutant intron 7 Exon 8

GGCUGUAAAGuuaagauau[a](http://www.ensembl.org/Homo_sapiens/ZMenu/TextSequence?db=core;factorytype=Location;g=ENSG00000101972;r=X:123094062-123556514;v=rs188012323;vf=48869732)uuu[a](http://www.ensembl.org/Homo_sapiens/ZMenu/TextSequence?db=core;factorytype=Location;g=ENSG00000101972;r=X:123094062-123556514;v=TMP_ESP_X_123171487;vf=57080215)uuuugaaaucagcagcucucaaauagucacuucguuguuccaccuaaagagauguuguuagcacuuuacaugGAGUUGUCACA

-G--C--K--V--K--I--Y--L--F--*

**Tumor 578 (3278-3_3296 del22)**

The mutation deletes the 3’ splice site of exon 32 and activates a cryptic 3’ splice site located within exon 32. RT-PCR products on an agarose gel show no normal sized band but instead a smaller band consistent with use of the cryptic splice site in exon 32 (i.e. exon 31 joined to most of exon 32 joined to exon 33, r.3278_3302 del25) and a larger band consistent with retention of intron 32 as well as use of the cryptic splice site. Use of cryptic site and intron retention were confirmed by sequencing.

DNA sequence with deleted nucleotides and cryptic splice site highlighted

123224382 tcaaaattag tgac[t](http://www.ensembl.org/Homo_sapiens/ZMenu/TextSequence?db=core;factorytype=Location;g=ENSG00000101972;r=X:123094062-123556514;v=TMP_ESP_X_123224396;vf=57080519)aaacc tc[g](http://www.ensembl.org/Homo_sapiens/ZMenu/TextSequence?db=core;factorytype=Location;g=ENSG00000101972;r=X:123094062-123556514;v=TMP_ESP_X_123224404;vf=57080523)tcgttaa ttttctttt[c](http://www.ensembl.org/Homo_sapiens/ZMenu/TextSequence?db=core;factorytype=Location;g=ENSG00000101972;r=X:123094062-123556514;v=TMP_ESP_X_123224421;vf=57080527) cag**CTGAAGA AAGTAGTAGT** 123224441

123224442 **AGTGACAGTA TGTGGTTAAG CAGAGAACAA ACACTGCACA CCCCTGTTAT GATGCAGACA** 123224501

Use of the cryptic 3’ splice site in exon 32 changes the reading frame and introduces a stop codon close to the site of the cryptic splice site - this means that both the longer and the shorter products have the same effect at the protein level, p.T1093fs.

Exon 31 Exon 32

CUUUCACUCAUAUGUGGUUAAGCAGA

-L--S--L--I--C--G--*-

**Tumor 670 (c.2775+2 delT)**

The mutation destroys the 5’ splice site of exon 28. The effect at the RNA level is skipping of exon 28. The final nucleotide of exon 27, c.2673, is joined to the first nucleotide of exon 29, c.2776. Exon 28 is 102 nt long, so the reading frame remains intact. At the RNA level, the change is r.2674_2775 del102, p.Y891_Q925 del.

Exon 27 Exon 29

CAGUAUAUGAAGCUUUUUAAUGAA

-Q--Y--M--K--L--F--N--E-

**Tumor 736 (2097-3 C>G)**

The mutation not only destroys the real exon 23 3’ splice site but also introduces a new cryptic 3’ splice site located 2 nucleotides upstream of the real site.

Wild type DNA sequence

123199962 gtcattaggc ttagcttttt aataaaactt aa[t](http://www.ensembl.org/Homo_sapiens/ZMenu/TextSequence?db=core;factorytype=Location;g=ENSG00000101972;r=X:123094062-123556514;v=rs11299777;v=rs35997278;vf=7649855;vf=12294264)[t](http://www.ensembl.org/Homo_sapiens/ZMenu/TextSequence?db=core;factorytype=Location;g=ENSG00000101972;r=X:123094062-123556514;v=rs35997278;vf=12294264)tttttt tttttttttt ttttttttta 123200021

123200022 cag**TGCCCAT GACCTTTCAA AGTGGGATTT ATTTGCTTGT AATTACAAAC TCTTGAAAAC** 123200081

Mutant DNA sequence with introduced cryptic splice site highlighted

123199962 gtcattaggc ttagcttttt aataaaactt aa[t](http://www.ensembl.org/Homo_sapiens/ZMenu/TextSequence?db=core;factorytype=Location;g=ENSG00000101972;r=X:123094062-123556514;v=rs11299777;v=rs35997278;vf=7649855;vf=12294264)[t](http://www.ensembl.org/Homo_sapiens/ZMenu/TextSequence?db=core;factorytype=Location;g=ENSG00000101972;r=X:123094062-123556514;v=rs35997278;vf=12294264)tttttt tttttttttt ttttttttta 123200021

123200022 gag**TGCCCAT GACCTTTCAA AGTGGGATTT ATTTGCTTGT AATTACAAAC TCTTGAAAAC** 123200081

The effect at the RNA level is that the final two nucleotides of intron 22 are inserted between the final nucleotide of exon 22, c.2096, and the first nucleotide of exon 23, c.2097: r.2096_2097 ins ag, p.N699fs.

Exon 22 Exon 23

UUUCAUAAagUGCCCAUGACCUUUCAAAGUGGGAUUUAUUUGCUUGUAAUUACAAACUCUUGA

-F--H--K--V--P--M--T--F--Q--S--G--I--Y--L--L--V--I--T--N--S--*-

**Tumor 860 (c.2533 G>A)**

The mutation in this tumor is in the final nucleotide of exon 26. It therefore has the potential to function by changing the primary amino acid sequence as the missense mutation p.D845N, by affecting splicing of exon 26 [Huang et al, 1993; Teraoka et al., 1999] or both.

In RT-PCR products from this tumor we detected a product that lacks exon 26 and a product which retains exon 26 but has the mutated nucleotide at position c.2533. c.2533 G>A is therefore presumed to act as both a splicing and a missense mutation.

Exon 26-retaining mutant RNA: r.2533 G>A, p.D845N

Exon 26-lacking mutant RNA: r.2359_2533 del175 : final nucleotide of exon 25, c.2358, is joined to the first nucleotide of exon 27, c.2534. p.A787fs

Exon 25 Exon 27

AAGGAACAGAUGGUCAGCAAGAGGAUGAAGCCAGUAAAAUUGAAGCUCUGCACAAGAGAAGAAAUUUACUUGCAGCAUUUUGUAAGCUAA

-K--E--Q--M--V--S--K--R--M--K--P--V--K--L--K--L--C--T--R--E--E--I--Y--L--Q--H--F--V--S--*-

**Tumor 945 (c.2256_2265+16 del26)**

The mutation deletes the exon 24 5’ splice site. This results in skipping of exon 24. The final nucleotide of exon 23, c.2184, is joined to the first nucleotide of exon 25, c.2266. Exon 24 is 81 nt long, so the product is in frame. r.2185_2265 del81, p.I729_K755del.

DNA Deleted nucleotides highlighted

123200202 aca[g](http://www.ensembl.org/Homo_sapiens/ZMenu/TextSequence?db=core;factorytype=Location;g=ENSG00000101972;r=X:123094062-123556514;v=rs12389430;vf=8670995)**ATTGTT ATTCACGCAC TGCAGTGTAC TCACTATGTA ATCCTTTGGC AACTTGCTAA** 123200261

123200262 **GATAACTGAA AGCAGCTCTA CAAAG**gtttg tg[g](http://www.ensembl.org/Homo_sapiens/ZMenu/TextSequence?db=core;factorytype=Location;g=ENSG00000101972;r=X:123094062-123556514;v=rs146973846;vf=37809792)tggttca gtagtg[t](http://www.ensembl.org/Homo_sapiens/ZMenu/TextSequence?db=core;factorytype=Location;g=ENSG00000101972;r=X:123094062-123556514;v=rs34494691;vf=10940971)ttt ta[t](http://www.ensembl.org/Homo_sapiens/ZMenu/TextSequence?db=core;factorytype=Location;g=ENSG00000101972;r=X:123094062-123556514;v=rs34397445;vf=10852228)tagacta 123200321

Exon 23 Exon 25

CCUGAGCAGGAGGACUUG

-P--E--Q--E--D--L-

**Tumor 961 (c.2026-1 G>T)**

The mutation destroys the 3’ splice site of exon 22. A cryptic 3’ splice site located within exon 22 is used instead.

DNA sequence with site of mutation and location of cryptic splice site highlighted.

123199722 atag**GGTGAA GAACCTGATG AAGATGATGC ATATCAGGTA TTGTCAACAT TGAAGAGGAT** 123199781

In the RNA product, the final nucleotide of exon 21, c.2025, is joined to nucleotide c.2033, within exon 22: r.2026_2032del7, p.G676fs.

Exon 21 Exon 22

CUGCAAGAGAACCUGAUGAAGAUGAUGCAUAUCAGGUAUUGUCAACAUUGAAGAGGAU

-L--Q--E--N--L--M--K--M--M--H--I--R--Y--C--Q--H--*

**Tumor 987 (c.1417-7 A>G)**

The mutation introduces an AG dinucleotide into intron 16, just upstream of the AG of the exon 17 3’ splice site. Although the sequence context of the new AG is AAG rather than the YAG of a consensus 3’ splice site, it nevertheless functions as a 3’ splice site in the mutant.

Wild type DNA sequence

123195042 tctaagacca cattgctctt tttaaatttt ag**TTACATGA GCATGCAGCA TACCTTGTGG** 123195101

Mutant DNA sequence, showing new cryptic 3’splice site.

123195042 tctaagacca cattgctctt tttaagtttt ag**TTACATGA GCATGCAGCA TACCTTGTGG** 123195101

In the RNA extracted from this tumor, the final nucleotide of exon 16, c.1416, is joined to the final six nucleotides of intron 16, followed by exon 17: r.1416_1417 ins uuuuag. At the protein level, this results in the insertion of two codons, the second of which is an in frame stop codon: p.L473fs.

Exon 16 intron 16 Exon 17

GAAAGUGAGuuuuagUUACAUGAGCAUGCAGCA

-E--S--E--F--*-

**Tumor 1046 (c.386-8 A>G)**

No RNA was available for this sample. The mutation is at the same site as the rare A>T variant rs199610347. The mutation introduces a cryptic 3’ splice site sequence, YAG, just upstream of the real exon 8 3’ splice site. Usually, the first AG residue downstream of the branchpoint/polypyrimidine tract will be used for splicing [Smith et al., 1993] and thus, as in this case, where two YAG sequences are available in tandem, the more 5’ is the one likely to be used.

wild type DNA sequence. w = rs199610347

123176382 a[c](http://www.ensembl.org/Homo_sapiens/ZMenu/TextSequence?db=core;factorytype=Location;g=ENSG00000101972;r=X:123094062-123556514;v=rs184515751;vf=45373413)accatata ttaacttctg acatttgca[w](http://www.ensembl.org/Homo_sapiens/ZMenu/TextSequence?db=core;factorytype=Location;g=ENSG00000101972;r=X:123094062-123556514;v=rs199610347;vf=54284448) atttcag**GAG TTGTCACAGC AGAAATGTTT** 123176441

123176442 **AGACATATGC AGAACTCTGA GATAATTCGA AAAATGACTG AAGAATTCGA TGAG**gtaact 123176501

mutant DNA sequence

123176382 a[c](http://www.ensembl.org/Homo_sapiens/ZMenu/TextSequence?db=core;factorytype=Location;g=ENSG00000101972;r=X:123094062-123556514;v=rs184515751;vf=45373413)accatata ttaacttctg acatttgca[g](http://www.ensembl.org/Homo_sapiens/ZMenu/TextSequence?db=core;factorytype=Location;g=ENSG00000101972;r=X:123094062-123556514;v=rs199610347;vf=54284448) atttcag**GAG TTGTCACAGC AGAAATGTTT** 123176441

123176442 **AGACATATGC AGAACTCTGA GATAATTCGA AAAATGACTG AAGAATTCGA TGAG**gtaact 123176501

The prediction for this sample is that the cryptic splice site in the mutant would be used in preference to the real 3’ splice site. r.(385_386 ins auuucag), p.(G129fs).

Exon 7 intron 7 Exon 8

GGCUGUAAAGauuucagGAGUUGUCACAGCAGAAAUGUUUAG

-G--C--K--D--F--R--S--C--H--S--R--N--V--*-

**Tumor 1142 (c.2534-4_2550 del21)**

The mutation deletes the 3’ splice site of exon 27. The RNA product skips exon 27. The last nucleotide of exon 26, c.2533, is joined to the first nucleotide of exon 28, c.2674. Exon 27 is 140 nucleotides long so its deletion is out of frame. r.2534_2673 del140, p.D845fs.

DNA with deletion highlighted

123210162 gtttgggact ttttctccag **ATGGTCAGCA AGAGGATGAA GCCAGTAAAA TTGAAGCTCT** 123210221

Exon 26 Exon 28

AAUAGUGCAGUAUUAUAAUGACUAUGGAGAUAUCAUCAAAGAAAC

-N--S--A--V--L--*-

**Tumor 1175 (c.1196+4 A>G)**

The mutation creates a new cryptic 5’ splice site 4 nucleotides downstream of the real exon 14 5’ splice site. In the RNA product, the cryptic site is used.

Wild type DNA sequence

123185202 **ATGTTGCAGT ACAAGCAATA AAATTACTCA CTCTTGTTTT ACA**gtaagta tgtatttgtt 123185261

Mutant DNA sequence showing location of cryptic 5’ splice site

123185202 **ATGTTGCAGT ACAAGCAATA AAATTACTCA CTCTTGTTTT ACA**gtaggta tgtatttgtt 123185261

In the RNA product, the final nucleotide of exon 14, c.1196, is separated from the first nucleotide of exon 15, c.1197, by the inclusion of the first four nucleotides of mutant intron 14: r.1196_1197 ins guag. The inserted intron nucleotides contain an in-frame stop codon and the effect is immediate termination with no frameshifted sequence, p.S400*.

Exon 14 intron 14 Exon 15

CUUGUUUUACAguagGAGUAGUGAAGAAGUUCUCACUGC

-L--V--L--Q--*-

**Tumor 1210 (c.2097-1 G>A)**

The mutation destroys the 3’ splice site of exon 23. This results in skipping of exon 23. The final nucleotide of exon 22, c.2096, is joined to the first nucleotide of exon 24, c.2185. Exon 23 is 88 nt long, so the resulting product is out of frame. r.2097_2184 del88, p.N699fs.

Exon 22 Exon 24

GCUUUUCAUAAAUUGUUAUUCACGCACUGCAGUGUACUCACUAUGUAAUCCUUUGGCAACUU

-A--F--H--K--L--L--F--T--H--C--S--V--L--T--M--*

**Tumor 1214 (c.2097-2 A>G)**

No RNA was available for this sample which has the same mutation as 94-10 (see below).

**Tumor 1231 (c.462 G>C)**

The mutation is in the final nucleotide of exon 8 and has the potential to act as a splicing mutation, a missense mutation or both. RT-PCR products from this sample were almost exclusively lacking exon 8. The level of residual exon 8-containing product was too low to be able to tell whether any contained the mutant nucleotide or whether it was all derived from contaminating normal tissue. The last nucleotide of exon 7, c.385, is joined to the first nucleotide of exon 9, c.463: r.386_462 del77. The reading frame is altered and the result is immediate termination without any frameshifted sequence: p.V130*.

Exon 7 Exon 9

GGCUGUAAAGGAUAGUGGAGAUU

-G--C--K--G--*-

**Tumor 1273 (c.2924+1 G>A)**

The mutation destroys the 5’ splice site of exon 29. The effect at the RNA level is skipping of exon 29. The final nucleotide of exon 28, c.2775, is joined to the first nucleotide of exon 30, c.2925. Exon 29 is 149 nucleotides long, so the resulting product is out of frame. r.2776_2924 del149, p.L926fs.

Exon 28 Exon 30

CUGCAACAGAGAUGGCAUAGAAUUUGCUUUUAAAGAGC

-L--Q--Q--R--W--H--R--I--C--F--*-

**Tumor 1345 (c.1535-40_1566 del72)**

No STAG2 RT-PCR products were generated using RNA from this sample. RT-PCR products were generated using a set of SDHA control primers indicating that the quality of the RNA was satisfactory. As the entire 3’ splice site of exon 18 is removed by the mutation, we would expect major perturbation of splicing, especially as no predicted cryptic splice sites are present in a useable orientation.

Exon skipping and use of local cryptic splice sites are the most common consequences of mutations affecting splice sites [Robberson et al., 1990]. Intron retention is another possible consequence. The introns flanking exon 18 are 428 and 1027 nucleotides in length respectively. Retention of either or both of these introns could make an RT-PCR amplicon that was too large to amplify efficiently in the experimental system used.

**Tumor 1383 (c.1017+1_1017+22 delins CATCTTAC)**

The mutation deletes the 5’ splice site of exon 12.

Wild type DNA sequence showing deleted nucleotides

123184122 **GTTATTTAAA ATATGTTGGT TGGACTATGC ATGATAAG**gt aagatgtgcc cttcagactg 123184181

123184182 ct[t](http://www.ensembl.org/Homo_sapiens/ZMenu/TextSequence?db=core;factorytype=Location;g=ENSG00000101972;r=X:123094062-123556514;v=TMP_ESP_X_123184184;vf=57080271)ctttcta cac[g](http://www.ensembl.org/Homo_sapiens/ZMenu/TextSequence?db=core;factorytype=Location;g=ENSG00000101972;r=X:123094062-123556514;v=rs201834695;vf=56483728)tcggcg tggctgtctg cacctctcat tcatgagtta tctcccagta 123184241

Mutant DNA sequence showing inserted nucleotides

123184122 **GTTATTTAAA ATATGTTGGT TGGACTATGC ATGATAAG** catcttac 123184181

123184182 ct[t](http://www.ensembl.org/Homo_sapiens/ZMenu/TextSequence?db=core;factorytype=Location;g=ENSG00000101972;r=X:123094062-123556514;v=TMP_ESP_X_123184184;vf=57080271)ctttcta cac[g](http://www.ensembl.org/Homo_sapiens/ZMenu/TextSequence?db=core;factorytype=Location;g=ENSG00000101972;r=X:123094062-123556514;v=rs201834695;vf=56483728)tcggcg tggctgtctg cacctctcat tcatgagtta tctcccagta 123184241

In the RNA product from the mutant, the real 5’ splice site of exon 12 is not available for use and instead a cryptic site located in intron 12 is used. This results in the insertion of 65 nucleotides derived from the mutant version of intron 12 being inserted between the final nucleotide of exon 12, c.1017, and the first nucleotide of exon 13, c.1018. RNA level effect: r.1017_1018 ins65, p.Q340fs.

Exon 12 Mutant intron 12 Exon 13

CAUGAUAAGcaucuuaccu[u](http://www.ensembl.org/Homo_sapiens/ZMenu/TextSequence?db=core;factorytype=Location;g=ENSG00000101972;r=X:123094062-123556514;v=TMP_ESP_X_123184184;vf=57080271)cuuucuacac[g](http://www.ensembl.org/Homo_sapiens/ZMenu/TextSequence?db=core;factorytype=Location;g=ENSG00000101972;r=X:123094062-123556514;v=rs201834695;vf=56483728)ucggcguggcugucugcaccucucauucaugaguuaucucccaCAAGGUGAA

-H--D--K--H--L--T--F--F--L--H--V--G--V--A--V--C--T--S--H--S--*-

**94-10 (c.2097-2 A>G)**

The mutation destroys the 3’ splice site of exon 23. By agarose electrophoresis, two RT-PCR products were observed: one slightly smaller than normal and one consistent with exon skipping. A cryptic, non-consensus 3’ splice site is present in exon 23.

DNA showing site of mutation and cryptic 3’splice site

123199962 gtcattaggc ttagcttttt aataaaactt aa[t](http://www.ensembl.org/Homo_sapiens/ZMenu/TextSequence?db=core;factorytype=Location;g=ENSG00000101972;r=X:123094062-123556514;v=rs11299777;v=rs35997278;vf=7649855;vf=12294264)[t](http://www.ensembl.org/Homo_sapiens/ZMenu/TextSequence?db=core;factorytype=Location;g=ENSG00000101972;r=X:123094062-123556514;v=rs35997278;vf=12294264)tttttt tttttttttt ttttttttta 123200021

123200022 cag**TGCCCAT GACCTTTCAA AGTGGGATTT ATTTGCTTGT AATT**[**W**](http://www.ensembl.org/Homo_sapiens/ZMenu/TextSequence?db=core;factorytype=Location;g=ENSG00000101972;r=X:123094062-123556514;v=rs112417641;vf=25527898)**CAAAC TCTTGAAAAC** 123200081

Use of the cryptic 3’ splice site is the major product. The final nucleotide of exon 22, c.2096, is joined to nucleotide c.2216 in exon 23, deleting the first 19 nucleotides of exon 23. r.2097_2115 del19, p.A700fs.

Exon 22 Exon 23

GCUUUUCAUAAUGGGAUUUAUUUGCUUGUAAUUACAAACUCUUGAAAAC

-A--F--H--N--G--I--Y--L--L--V--I--T--N--S--*

A minor product skips exon 23 altogether. The final nucleotide of exon 22, c.2096, is joined to the first nucleotide of exon 24, c.2185: r.2097_2184 del88, p.N699fs.

Exon 22 Exon 24

GCUUUUCAUAAAUUGUUAUUCACGCACUGCAGUGUACUCACUAUGUAAUCCUUUGGCAACUU

-A--F--H--K--L--L--F--U--H--C--S--V--L--U--M--*

**UM-UC14 (c.2026-1 G>T)**

The mutation destroys the exon 22 3’ splice site. At the RNA level, use of local cryptic 3’ splice sites located in intron 21 and exon 22 is observed.

DNA sequence with site of mutation highlighted.

123199602 tgaatgacaa agttcatttg tgggttttgt tatgttattt ttcatttttc aaggtaatag 123199661

123199662 tgacattttg atag[c](http://www.ensembl.org/Homo_sapiens/ZMenu/TextSequence?db=core;factorytype=Location;g=ENSG00000101972;r=X:123094062-123556514;v=rs189298207;vf=50163430)ctagg agttttcact [t](http://www.ensembl.org/Homo_sapiens/ZMenu/TextSequence?db=core;factorytype=Location;g=ENSG00000101972;r=X:123094062-123556514;v=rs10218354;vf=6749577)tgatggttc ttaatgtata attaatattt 123199721

123199722 atag**GGTGAA GAACCTGATG AAGATGATGC ATATCAGGTA TTGTCAACAT TGAAGAGGAT** 123199781

123199782 **CACTGCTTTT CATAA**gtaag ttgaattttg aagttcctgt tttcttaaat ctgtagaaat 123199841

UM-UC14 produces two abnormal RNA products. The smaller product joins the final nucleotide of exon 21, c.2025, to nucleotide 2033 in exon 22. At the RNA level, this change is r.2026_2032 del7 which results in p.G676fs.

Exon 21 Exon 22

UUUCUGCAAGAGAACCUGAUGAAGAUGAUGCAUAUCAGGUAUUGUCAACAUUGA

-F--L--Q--E--N--L--M--K--M--M--H--I--R--Y--C--Q--H--*-

The larger product joins the final nucleotide of exon 21 to nucleotide c.2026-71 in intron 21. At the RNA level this is r.2025_2026 ins71, p.G676fs.

Exon 21 Intron 21

UUUCUGCAAGAGguaauagugacauuuuga

-F--L--Q--E--V--I--V--T--F--*-

In tumor 961, which has the same mutation as UM-UC 14, only the exon 22 cryptic 3’ splice site is used (see above). It is possible that this may reflect differences within the splicing machinery between the two samples, which may or may not be tumor-associated; or may be associated with the presence of further *STAG2* sequence changes, either germline or somatic, outside of the region screened.

**VM-CUB-I (c.2674-6_2686 dup)**

The mutation duplicates sequence encompassing the exon 28 3’ splice site. Although this apparently creates two 3’ splice sites in tandem, neither of these is used and what is observed in the RNA sample is skipping of exon 28. The last nucleotide of exon 27, c.2673, is spliced to the first nucleotide of exon 29, c.2776. Exon 28 is 102 nucleotides long, so the reading frame remains intact. At the RNA level, the change is r.2674_2775 del102, p.Y892_Q925 del.

DNA sequence with duplicated nucleotides highlighted

123211782 ctaatttgta caaatttctt tatag**TATTA TAATGACTAT GGAGATATCA TCAAAGAAAC** 123211841

Mutant DNA sequence

123211782 ctaatttgta caaatttctt tatag**TATTA TAATGACT**t tatag**TATTA TAATGACTAT GGAGATATCA**

Exon 27 Exon 29

CAGUAUAUGAAGCUUUUUAAUGAA

-Q--Y--M--K--L--F--N--E-

**VM-CUB-III (c.289-2 A>G)**

The mutation destroys the 3’ splice site of exon 7. A cryptic 3’ splice site within exon 7 is used instead. The consequence is the removal of 10 nucleotides from the 5’ end of exon 7. The final nucleotide of exon 6, c.288, is joined to nucleotide c.299 within exon 7. r.289_298 del10. p.S97fs.

DNA sequence showing position of mutation and cryptic 3’ splice site

123171342 aagctaatga t[t](http://www.ensembl.org/Homo_sapiens/ZMenu/TextSequence?db=core;factorytype=Location;g=ENSG00000101972;r=X:123094062-123556514;v=TMP_ESP_X_123171353;vf=57080208)ttattttt ttccctctgc t[g](http://www.ensembl.org/Homo_sapiens/ZMenu/TextSequence?db=core;factorytype=Location;g=ENSG00000101972;r=X:123094062-123556514;v=rs201406127;vf=56052712)tag**TCGGT GGTAGATGAT TGGATAGAAT** 123171401

Exon 6 Exon 7

GCUAUGCAGAUGAUUGGAUAGAAU

-A--M--Q--M--I--G--*

Huang C-H, Reid M, Daniels G, Blumenfeld OO. (1993). Alteration of splice site selection by an exon mutation in the human glycophorin A gene. J. Biol. Chem. **268:** 25902-25908.

Robberson BL, Cote GJ & Berget SM. (1990). Exon definition may facilitate splice site selection in RNAs with multiple exons. Mol. Cell. Biol. **10:** 84-94.

Smith, CWJ, Chu, TT & Nadal-Ginard B. (1993). Scanning and competition between AGs are involved in 3’ splice site selection in mammalian introns. Mol. Cell. Biol. **13:** 4939-4952.

Teraoka SN, Telatar M, Becker-Catania S, Liang T, Onengut S, Tolun A et al. (1999). Splicing defects in the ataxia-telangiectasia gene *ATM*: underlying mutations and consequences. Am. J. Hum. Genet. **64:** 1617-1631.
