# Supplementary material for: Frequent inactivating mutations of STAG2 in bladder cancer are associated with low tumour grade and stage and inversely related to chromosomal copy number changes
Source: Hum Mol Genet. 2013 Nov 22;23(8):1964–74. doi: 10.1093/hmg/ddt589 (PMC3959811; doi:10.1093/hmg/ddt589)
Supplement: Supplementary Data [file supp_ddt589_ddt589supp_table2.docx]

**Supplementary Table 2. Clinico-pathological characteristics of 47 bladder tumor-derived cell lines.**

| **Cell line** | **Type** | **Grade** | **Stage** | **Origin** | **Reference** |
| --- | --- | --- | --- | --- | --- |
| 253J | TCC | G4 | T4 | Lymph node metastasis, | 1 |
| 5637 | TCC | NR | NR | Bladder, primary | 2 |
| 639V | TCC | G3 | "small" | Ureter | 3 |
| 647V | TCC | G2 | "malignant" "stage B" | Bladder, primary | 3,4 |
| 92-1 | TCC | G3 | T4 | Bladder | 5 |
| 94-10 | TCC | G2-3 | T3 | Bladder, papillary | 5 |
| 96-1 | TCC | G2-3 | T3 | Bladder | 5 |
| 97-1 | TCC | G1-2 | T1/T2 | Bladder, papillary | 5 |
| 97-18 | TCC | G3 | T2 | Bladder | 6 |
| 97-24 | TCC | G3 | T3 | Bladder | 6 |
| 97-7 | TCC | G2-3 | T1 | Bladder | 6 |
| BC3C | TCC | G4 (Bergkvist) | "stage C" (UICC) | Bladder | 7 |
| BFTC905 | TCC | G3 | "stage D1"(Jewett) | Bladder, primary, papillary | 8 |
| BFTC909 | TCC | G3 | T4N2M1 | Renal pelvis | 8 |
| CAL29 | TCC | G4 | T2 | Bladder, primary, invasive | 9 |
| DSH1 | TCC | G2 | T1a | Bladder, recurrence, papillary | 10 |
| HCV29 |  |  |  | Non-malignant ureteric epithelium of a patient with bladder cancer | Fogh 1971 (unpublished work) |
| HT1197 | TCC | G4 | T2 at least | Bladder, recurrence | 11 |
| HT1376 | TCC | G3 | Invasive | Bladder | 11 |
| J82 | TCC | G3 | T3 | Bladder, primary, papillary with solid areas | 12 |
| JMSU1 | TCC | NR | NR | Established from malignant ascitic fluid of a patient with bladder cancer | 13 |
| JO'N | TCC | NR | NR |  |  |
| KU-19-19 | TCC | G3 | T3b |  | 14 |
| LUCC1 | TCC | G3 | T3bN1 | Bladder, recurrence | Pitt, unpublished |
| LUCC2 | TCC | G2 | T2a | Bladder, recurrence | Pitt, unpublished |
| LUCC3 | TCC | G3 | T2 at least | Bladder, primary | Pitt, unpublished |
| LUCC4 | TCC | G3 | T2 at least | Bladder, primary | Pitt, unpublished |
| LUCC5 | TCC | G3 | T2 at least | Bladder, primary | Pitt, unpublished |
| LUCC6 | TCC | G3 | Ta | Renal pelvis, primary, superficial | Pitt, unpublished |
| LUCC7 | TCC | G3 | T3 | Renal pelvis, primary | Pitt, unpublished |
| LUCC8 | TCC | G2 | Ta | Bladder, primary, papillary | Pitt, unpublished |
| LUCC9 | TCC | G3 | T3b | Bladder, plasmacytoid variant with focal neuroendocrine differentiation | McPherson, unpublished |
| MGH-U3 | TCC | G1 | "non-invasive" | Bladder, recurrence, papillary | 15 |
| RT112 | TCC | G2 | NR | Bladder, primary, papillary | 16 |
| RT4 | TCC | G1 | T2 | Bladder, recurrence | 17 |
| SCaBER | SCC | "moderately differentiated" |  | Bladder, primary | 18 |
| SD | TCC | NR | NR | Bladder, primary | 19 |
| SW1710 | TCC | G3 | NR | Bladder, papillary | 20 |
| SW780 | TCC | G1 | NR | Bladder, primary, papillary | 2,21 |
| T24 | TCC | G3 | NR | Bladder | 22 |
| TCCSUP | TCC | G4 | NR | Bladder | 23 |
| U-BLC1 | TCC | G3 | T2 at least | Bladder, primary | 24 |
| UM-UC3 | TCC | NR | NR | Bladder | 25 |
| UM-UC14 | TCC | NR | NR | Renal pelvis | 26 |
| VM-CUB-1 | TCC | NR | NR | Bladder, primary | 21 |
| VM-CUB-2 | TCC | NR | NR | Lymph node metastasis | 21 |
| VM-CUB-3 | TCC | NR | NR | Bladder primary | 21 |

TCC – Transitional Cell Carcinoma; SCC – Squamous Cell Carcinoma; NR – Not Recorded

1. Elliott, A.Y. *et al. J. Natl. Cancer Ins.* **53**, 1341-1349 (1974).
2. Fogh, J. *Natl. Cancer Inst. Monogr.* **49**, 5-9 (1978).
3. Elliott, A.Y., Bronson, D.L., Stein, N. & Fraley, E.E*. Cancer Res.* **36**, 365-369 (1976).
4. Elliott, A.Y., Bronson, D.L., Cervenka, J., Stein, N. & Fraley, E.E. *Cancer Res*. **37**, 1279-1289 (1977).
5. Yeager, T.R. *et al. Genes Dev*. **12**, 163-174 (1998).
6. Sarkar, S. *et al*. *Cancer Res*. **60**, 3862-3871 (2000).
7. Pratsinis, H., Saetta, A., Gagos, S., & Davaris, P. *In Vitro Cell Dev. Biol. Anim*. **34**, 722-728 (1998).
8. Tzeng, C.C. *et al. Anticancer Res*. **16**, 1797-1804 (1996).
9. Cattan, N. *et al. Br. J. Cancer* **85**, 1412-1417 (2001).
10. Williams, S. *et al. Genes Chromosomes Cancer* **34**, 86-96 (2002).
11. Rasheed, S., Gardner, M.B., Rongey, R.W., Nelson-Rees, W.A. & Arnstein, P. *J. Natl. Cancer Inst.* **58**, 881-90 (1977).
12. O'Toole, C., Price, Z.H., Ohnuki, Y. & Unsgaard, B. *Br. J. Cancer* **38**, 64-76 (1978).
13. Morita, T., Shinohara, N., Honma, M. & Tokue A. *Urol. Res*. **23**, 143-149 (1995).
14. Tachibana, M. *et al. Cancer Res*. **55**, 3438-3443 (1995).
15. Lin C.W., Lin J.C. & Prout G.R. Jr. *Cancer Res.* **45**, 5070-5079 (1985).
16. Marshall, C.J., Franks, L.M. & Carbonell, A.W. *J .Natl. Cancer Inst*. **58**, 1743-1751 (1977).
17. Rigby, C.C. & Franks, L.M. *Br. J. of Cancer* **24**, 746-754 (1970).
18. O'Toole, C., Nayak, S., Price, Z., Gilbert, W.H. & Waisman, J. *Int. J. of Cancer* **17**, 707-714 (1976).
19. Paulie, S., Hansson, Y., Lundblad, M.L. & Perlmann, P. *Int. J. Cancer* **31**, 297-303 (1983).
20. Kyriazis, A.A., Kyriazis, A.P., McCombs, W.B. 3rd & Peterson W.D. Jr*. Cancer Res.* **44**, 3997-4005 (1984).
21. Williams, R.D. *Invest. Urol.* **17**, 359-363 (1980).
22. Bubenik, J. *et al. Int. J. Cancer* **11**, 765-773 (1973).
23. Nayak, S.K., O'Toole, C. & Price, Z.H. *Br. J. Cancer* **35**, 142-51 (1977).
24. Bruch, J*. et al. Int. J. Cancer* **80**, 903-910 (1999).
25. Grossman, H.B., Wedemeyer, G., Ren, L., Wilson, G.N. & Cox, B. *J. Urol*. **136**, 953-959 (1986).
26. Sabichi, A. *et al*. *J. Urol.* **175**, 1133-1137 (2006).
