# Supplementary material for: Frequent inactivating mutations of STAG2 in bladder cancer are associated with low tumour grade and stage and inversely related to chromosomal copy number changes
Source: Hum Mol Genet. 2013 Nov 22;23(8):1964–74. doi: 10.1093/hmg/ddt589 (PMC3959811; doi:10.1093/hmg/ddt589)
Supplement: Supplementary Data [file supp_ddt589_ddt589supp_table3.docx]

**Supplementary Table 3. Primers used for analysis of STAG2**

**1. PCR/HRM primers**

| Exon | Forward^†^ | Reverse^†^ |
| --- | --- | --- |
| 4 | TCCGAATATTTTTGGTGCATT | CCCCATTTTGTGGAATAAACA |
| 5 | TTGTAATGAGTTAACCAAGCCTTT | AGATCTTAGGAAACAAAATAAAGTCAA |
| 6 | TGTAGCTGTGTTTTGAACTCTCAAG | ACAAATGAACTGGGAAGAACAACA |
| 7 | ggccTCTTGAATTTTATGACTTTATCAGACT | TAGGTGGAACAACGAAGTGA |
| 8 | CACCATATATTAACTTCTGACATTTGC | GCCCAGCCTAATGCTTACAA |
| 9 | TGCATTCTAAATGAAATTGCTG | TCAAATCTAAGACAATATGCAGAAAA |
| 10 | TGTGTCTGTTAGATTAGTTTCACCA | CAAGTTGGTCACACAATAGCC |
| 11 | GGTGTTCATTTGGTTGTCTTCC | TCCCTACACCACGAAATATGC |
| 12 | TTATGCATCGTTTTTCCTTCC | CATGAATGAGAGGTGCAGACA |
| 13 | GAGTTTTAATGCATTGTCTCATCT | ggccAAATGACAAGTTATTTGTAAATTCTTA |
| 14 | ACCAGTCGGTTCAAGGTTAG | AGTGCAAATATGCAACAAATACA |
| 15 | AGGACGTTACTAAAAGCACCTG | ccggTGAAAGTATGCCTTAGAAAATGAGT |
| 16 | ggccTTTTTGTTGTTGTTGTCGTAAAAT | TGCCTCATTTTAACCCCTTT |
| 17 | TGTCTAAGACCACATTGCTCTTTTT | ggccAAAAGGACAAATAAATGGAATGAGTAA |
| 18 | CAAGTGGCATATAGGGAGAAGAA | ACTGAAAAAGGAGAAATGGAGTTT |
| 19 | CACTTAACAGTGCTAATGGGCTTA | TCTGTGAGGCATTTAGGGAAA |
| 20 | TTCCCTAAATGCCTCACAGAA | CAAACGCTATGATATAATGAACTGAA |
| 21 | cgcgCCTATCATATATGCCTTAGTTTTGATG | AGGGGCTCAACTGACAAAAT |
| 22 | CATTTTGATAGCCTAGGAGTTTTCA | CATAACCCACAAAACATGCAA |
| 23 | CCAAAACAATTGTCATTAGGCTTAG | AAAAGTCACAAGACTTCACTTTTCAA |
| 24 | TCTTTGAAAAGTGAAGTCTTGTGA | TGTGAGTTTGCTGAAAACAGTTA |
| 25 | ccggTGAAAACATTTTAATGAAATGCTGAA | GGTTGCTTACAAAAAGATTACCATC |
| 26 | GGCAGTTAGTGAGAAACCTTGG | ccggTTTGATTTTATGGTGGACACAGA |
| 27 | CAAGATGCTTAATGTTTGGGACT | GGCAGCCATGCATAAAAACT |
| 28 | cgcgAACCTAAAATTTGTCTTATTGTCAAGT | TTTCAACTGCTACCTTATTTTGC |
| 29 | AACATGCTTTCTTTCTTTCCAA | TGAGAGCTGAATAAAATATCTGGA |
| 30 | TGAGTGAAATTTCCTAAGTTATTGAC | GCCCTTAAGAATCCGAAAAT |
| 31A* | gcgcTATGCCTATGCTCGCACAAC | CGGCTGCTGATTCCACTAA |
| 31B* | TTTGCTAGCTGGTGGTGATG | GAACCTTAATGACAATTCAGTTGG |
| 32 | CCTCGTCGTTAATTTTCTTTTC | ccggGGAGAATTTTATTCCTCTTTTAACTT |
| 33 | cgcgTTAAGTTAAAAGAGGAATAAAATTCTCC | AGTGAACTTCCTAGACTCAGACAA |
| 34 | CATTAGTTCAGATCTTGACTTTGTT | gcgcAATATCCAACATAATTTTCAAAAGA |
| 35 | CATAGACCATGTGAAGAACTTGG | TTTTGCAATCCTAGGGTACATTT |
| 36 | CGAGATTTTCTCCCCTCTCTC | TCCACTTAGAAAATGACTTCACCA |

*exon 31 was amplified in two overlapping PCRs.

†nucleotides shown in lowercase red are short GC clamps added to improve the melting curve of the PCR product.

**2. Sequencing primers**

Unless indicated below, PCR primers were also used for sequencing.

| Exon | Direction | Sequence |
| --- | --- | --- |
| 6 | reverse | CTGGGAAGAACAACATAAATC |
| 13 | forward | TTTTTTTAGCAAGGTGAAGT |
| 18 | forward | TTTTTTTAGCACTAACAGAT |
| 20 | forward | TATAATGCTTTCTTATTGTG |
| 21 | forward | TATTATTTTGCAGCATTTGG |
| 23 | forward | TTTTTTTACAGTGCCCATG |
| 26 | forward | TTTTTTTTAAATAGGCCTTC |
| 28 | reverse | GTGTTAGCTAAAGTTGGTAC |
| 31B | forward | GTGGTGATGATGACACCATG |

**3. FFPE-PCR primers**

The following primers were used for PCR of specific mutations from FFPE sections of individual tumours. If the PCR primer was M13-tagged then the cognate M13 primer was used for sequencing; otherwise the PCR primer was also used for sequencing.

| Exon | Tumor | Forward | Reverse |
| --- | --- | --- | --- |
| 7 | 359 | CGAGATATAGCACTTCTTGACCTT | use HRM primer |
| 17 | 1072 | GCTTGTTACTGGAAGAGCCACT* | use HRM primer |
| 18 | 454 | GCAAGAGAGTGCTCTGATTGAA | TTGGCATACCCTTTTTCCTG* |
| 19 | 1298 | GGTGCTTACAGCAAAGGAGA* | ACTTTTGCTAATAACTGAGGAAGG |
| 20 | 1028 | TTGCCTCAGTACTTTGATTTGG* | use HRM primer |
| 21 | 1338 | GATATTTCAAGAAGTCAACTGATAGAT | use HRM primer |
| 24 | 271 | TCTTTGAAAAGTGAAGTCTTGTGA* | GCAAGTTGCCAAAGGATTACA |
| 29 | 989 | AACTTGCTCGACGTTTTGCT | TGAGAGCTGAATAAAATATCTGGAG* |
| 31 | 500 | CCCACAGATTTCTGTATCAAAGC* | GTGGAAGCCACACATCCTCT* |

*primer has M13 tag. Forward primers were tagged with M13F (tgtaaaacgacggccagt) and reverse primers with M13R (caggaaacagctatgacc).
